# The TGFβ2‐Snail1‐miRNA_TGFβ2_ Circuitry is Critical for the Development of Aggressive Functions in Breast Cancer

**DOI:** 10.1002/ctm2.1558

**Published:** 2024-02-01

**Authors:** Liyun Luo, Ning Xu, Weina Fan, Yixuan Wu, Pingping Chen, Zhihui Li, Zhimin He, Hao Liu, Ying Lin, Guopei Zheng

**Affiliations:** ^1^ Affiliated Cancer Hospital and Institute of Guangzhou Medical University State Key Laboratory of Respiratory Disease Guangzhou China

**Keywords:** breast cancer, chromatin configuration, epithelial‐mesenchymal transition, miRNAs, TGFβ2

## Abstract

There have been contradictory reports on the biological role of transforming growth factor‐βs (TGFβs) in breast cancer (BC), especially with regard to their ability to promote epithelial‐mesenchymal transition (EMT). Here, we show that TGFβ2 is preferentially expressed in mesenchymal‐like BCs and maintains the EMT phenotype, correlating with cancer stem cell‐like characteristics, growth, metastasis and chemo‐resistance and predicting worse clinical outcomes. However, this is only true in ERα^−^ BC. In ERα^+^ luminal‐type BC, estrogen receptor interacts with p‐Smads to block TGFβ signalling. Furthermore, we also identify a microRNAs (miRNAs) signature (miRNAs_TGFβ2_) that is weakened in TGFβ2‐overexpressing BC cells. We discover that TGFβ2‐Snail1 recruits enhancer of zeste homolog‐2 to convert miRNAsTGFβ2 promoters from an active to repressive chromatin configuration and then repress miRNAs_TGFβ2_ transcription, forming a negative feedback loop. On the other hand, miRNAs_TGFβ2_ overexpression reverses the mesenchymal‐like traits in agreement with the inhibition of TGFβ2‐Snail1 signalling in BC cells. These findings clarify the roles of TGFβ2 in BC and suggest novel therapeutic strategies based on the TGFβ2‐Snail1‐miRNAs_TGFβ2_ loop for a subset type of human BCs.

## Graphical abstract

1. TGFβ2 was the major isoform overexpressed in TNBC.
2. TGFβ2 maintains the EMT and aggressive phenotype of TNBC.
3. ERα interacts with p‐Smads to block TGFβ signaling.
4. Snail1 recruits EZH2 to repress miRNAs targeting TGFβ2.

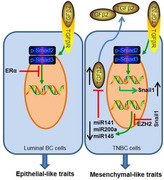

## 1 INTRODUCTION

Breast cancer (BC) is a common heterogeneous solid tumour,^1^, ^2^ that can be divided into four major subtypes: luminal A and luminal B subtypes, ERBB2 subtypes, basal‐like subtypes.^3^, ^4^ Basal‐like BC (BLBC) is a more aggressive disease, is more prone to recurrence and systemic metastasis, and has a higher recurrence rate in younger women than other types.^5^, ^6^, ^7^, ^8^, ^9^ The term “basal‐like” is derived from the fact that the molecular profile of this subgroup of tumours is similar to that of undifferentiated breast stem/progenitor cells in normal breast basal/myoepithelial cells (called basal‐like cells).^8^ BLBC is negative for ERα, PR and HER2 expression but positive for the basal markers CK5/6 and CK14; it is also known as triple‐negative BC (TNBC).^9^ The lack of effective targeted therapy and the adverse reactions of TNBC to standard chemotherapy regimens often lead to rapid patient death. Intriguingly, TNBC often activates the epithelial‐mesenchymal transition (EMT) program, which is a process of cell dedifferentiation that promotes the acquisition of cell pluripotency during embryonic development, inflammatory response, tissue fibrosis and cancer metastasis.^10^, ^11^, ^12^, ^13^, ^14^, ^15^ EMT reduces the adhesion between tumour cells and increases the ability of tumour cell motility. EMT endows tumour cells with similar properties to cancer stem cells (CSCs) and is prone to resistance during drug therapy.^16^, ^17^, ^18^, ^19^ Moreover, there are a large number of circulating tumour cells containing EMT and CSC markers in the blood samples of BC patients.^20^, ^21^, ^22^, ^23^, ^24^, ^25^, ^26^, ^27^, ^28^ Thus, clarification of the molecular circuitry that regulates EMT should facilitate major advances in developing therapeutic approaches to treat and prevent this deadly disease.

Transforming growth factor‐β (TGFβ) is considered to be a multifunctional cytokine that induces EMT and plays a key role in embryonic development and tissue homeostasis. TGFβ also regulates inflammation, and immune responses, and suppresses epithelial cell proliferation.^29^, ^30^ TGFβ functions as a tumour suppressor by inhibiting cell growth and increasing apoptosis at the early stages of tumour growth, whereas at the late stage, it functions as a tumour promoter to induce EMT, increase invasion and metastasis, suppress immune response and facilitate host‐cancer cell interactions.^29^, ^30^ TGFβ binds to type I and type II serine‐threonine kinase receptors (TβRI and TβRII).^31^ Then TβRII transphosphorylates TβRI, which activates Smad2 and Smad3 to form complexes with Smad4 and then undergoes nuclear translocation, regulating the transcription of downstream target genes under the synergistic action of multiple transcription factors and transcription coactivators, including Snail1, which is a key EMT transcription factor that promotes EMT in various cellular systems.^32^ E2 and TGFβ regulate the expression of ER‐α and Smad2/3 inhibits EMT activation in glioblastoma multiforme.^33^ Intriguingly, there are three different isoforms of TGFβ in the human genome: TGFβ1, TGFβ2 and TGFβ3. It remains completely unclear which isoform of TGFβ induces and maintains EMT in TNBC and how these TGFβs are regulated during tumour progression and metastasis. Snail1 is a transcription factor that contains a zinc finger and participates in the regulation of EMT during tumour occurrence and development.^34^ Snail1 overexpression induces tumour growth, tumour metastasis and BC stem cell (CSC)‐like properties.^35^, ^36^ Snail1 forms a complex with Smad3/4, occupying the promoters of the CAR and E‐cadherin genes and promoting TGFβ‐mediated EMT in BC.^37^

In the present study, we found that TGFβ2 was the major isoform that overexpressed in TNBC cell lines and tumour samples both in vitro and in vivo. Its expression is required to maintain the mesenchymal traits and aggressive behaviours observed in TNBC. In addition, this TGFβ2/Smad signalling pathway increases the expression of Snail1, which interacts with and recruits enhancer of zeste homolog‐2 (EZH2) to silence the expression of a particular microRNA (miRNA) miRNAs_TGFβ2_ (contains miR‐145, miR‐200a and miR‐141), a major endogenous TGFβ2 suppressor. This TGFβ2/Smad‐Snail1/EZH2‐miRNAs_TGFβ2_ feedforward circuitry is only operated in TNBC, not in luminal subtype BC because ERα interrupts this circuitry by interacting with Smad and blocking Snail1 expression in luminal BC.

## 2 MATERIALS AND METHODS

### 2.1 Patient samples

Serum samples were collected from 139 BC patients and 30 non‐cancerous healthy donors served as controls. The 72 fresh BC tissues and adjacent normal breast tissues were collected from BC patients, snap‐frozen and stored in liquid nitrogen. Serum samples and frozen fresh tissue samples were collected prior to any therapeutic procedures such as chemotherapy and radiotherapy at the Affiliated Cancer Hospital and Institute of Guangzhou Medical University. The cohort of paraffin‐embedded human tissues was obtained from 117 BC patients and 15 patients undergoing breast reduction surgery at Affiliated Cancer Hospital and Institute of Guangzhou Medical University. The other cohort of paraffin‐embedded human tissues were obtained from 104 BC patients and 13 patients undergoing breast reduction surgery at the Sun Yat‐Sen University Cancer Center.

### 2.2 Cell culture and treatment

Related cell lines were cultured as described previously.^68^ Stable transfection vectors with knockdown or expression were established by transfecting related cell lines with shRNAs (psi‐LVRU6GP vectors) or overexpression constructs (pLEX‐MCS) and selected stable clones with puromycin. The related cells were treated with 10 ng/mL TGFβ1 or TGFβ2.

### 2.3 miRNA in situ hybridization assay

The miRNAs_TGFβ2_ expression in BC samples was detected by in situ hybridization (ISH) with a kit from Exiqon according to the manufacturer's instructions. The methods used are described in the Supplemental Experimental Procedures.

### 2.4 Xenograft studies

All animal work was done in accordance with a protocol approved by the Institutional Animal Care and Use Committee at Guangzhou Medical University. Related cells (2 × 10^5^) were injected into the BALB/c nude mice (4–6 week‐old). Thirteenth day after cancer cell transplantation, the mice were injected intraperitoneally with 5‐Fu (30 mg/kg) or Paclitaxel (15 mg/kg). Treatment was performed every 3 days for a total of six cycles. Tumor sizes were measured for the whole duration of the experiment. Tumours were harvested and weighed at the experimental endpoint.

### 2.5 Enzyme‐linked immunosorbent assay, western blot, quantitative reverse transcription‐polymerase chain reaction, immunohistochemistry, immunofluorescence, transwell assay, MTS assay, flow cytometry, mammosphere assay, Luciferase reporter assay, co‐immunoprecipitation, chromatin immunoprecipitation and chromatin immunoprecipitation‐quantitative polymerase chain reaction

The methods used are described in the Supporting Information experimental procedures.

### 2.6 Statistics

Statistical analyses were performed using SPSS version 16.0 (SPSS) and GraphPad Prism 6. Survival curves were plotted using the Kaplan‐Meier method and compared using the log‐rank test. *p* < .05 was considered statistically significant. For most of the in vitro and animal experiments, data are presented as mean ± SEM and student's *t*‐tests were used to calculate the *p*‐value.

### 2.7 Study approval

Animal care and experiments were conducted in accordance with a protocol approved by the Institutional Animal Care and Use Committee at Guangzhou Medical University. Tissue and serum samples in this study were reviewed and approved by the Ethics Committees of the Affiliated Cancer Hospital of Guangzhou Medical University and the Sun Yat‐Sen University Cancer Center with informed consent from patients. The study was conducted in accordance with the Declaration of Helsinki.

## 3 RESULTS

### 3.1 TGFβ2 but not TGFβ1 or TGFβ3 is overexpressed in TNBC

**FIGURE 1:**
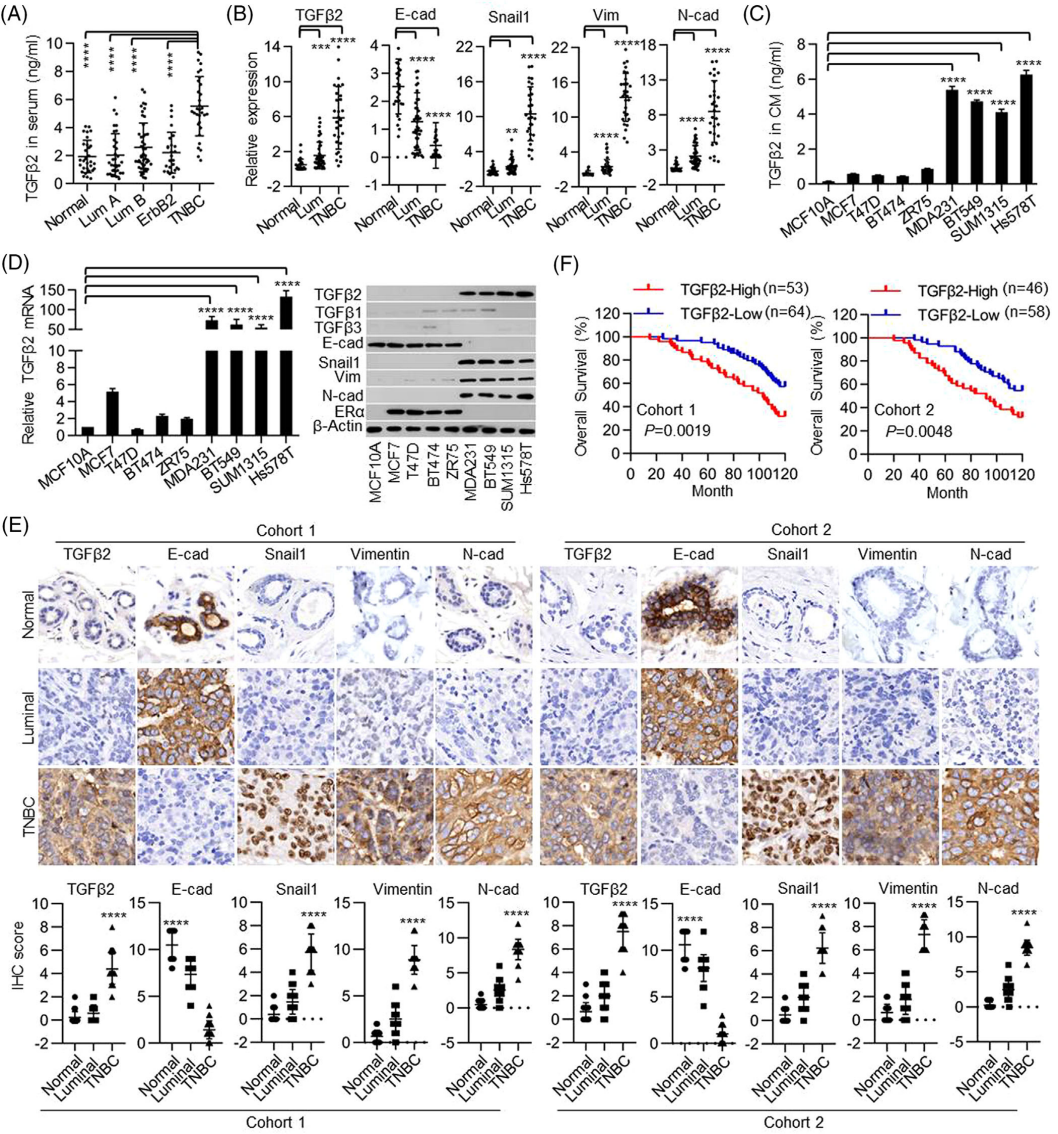
Transforming growth factor‐β2 (TGFβ2) expression pattern in breast cancer (BC). (A) TGFβ2 protein level in the serum of BC patients or healthy controls was determined with enzyme‐linked immunosorbent assay (ELISA). Differences between the two groups were assessed by a two‐way analysis of variance (ANOVA) test. (B) The relative expression of TGFβ2, E‐cadherin, Snail1, Vimentin and N‐cadherin at mRNA levels were examined by quantitative reverse‐transcription polymerase chain reaction (qRT‐PCR) in Normal, triple‐negative BC (TNBC) and luminal BC. Differences between the two groups were assessed by a two‐way ANOVA test. (C) TGFβ2 protein levels in cell culture medium were detected by ELISA. MCF10A served as the control group. Two‐sided student's t‐tests were performed. (D) TGFβ2 mRNA and protein levels in cell lines were evaluated by qRT‐PCR (left) and western blot (right), respectively. MCF10A served as the control group. Two‐sided student's t‐tests were performed. (E) Representative IHC staining for TGFβ2, E‐cadherin, Snail1, Vimentin and N‐cadherin in the two selected cohorts of breast cancer tissues (20×). Differences between the two groups were assessed by a two‐way ANOVA test. (F) Kaplan‐Meier analysis estimated overall survival based on TGFβ2 expression. The histologic score was used to evaluate the expression of TGFβ2. Survival curves were plotted using the Kaplan‐Meier method and compared using the log‐rank test. *p* < .05 was considered statistically significant. The data are presented as the mean ± SEM of at least three independent experiments. **, *p* < .01, ***, *p* < .005, ****, *p* < .0005.

To fully understand the critical role of TGFβs in BC, we measured TGFβ1, TGFβ2 and TGFβ3 protein levels in serum samples from 139 BC patients without any pretreatment and 30 healthy women. We found that the TGFβ2 level was significantly higher in TNBC (also commonly referred to as BLBC) patients than in luminal subtype (ERα positive) BC patients and healthy controls (Figure 1A). Although the TGFβ1 level in all BC subtypes was slightly higher than that of healthy controls, there was no significant difference between luminal and TN subtypes. A similar observation was also found for TGFβ3 (Figure S1A,B). From the 139 patients whose serum was analyzed, 43 luminal subtypes and 29 TNBC contained fresh‐frozen matched tumour samples and adjacent normal breast tissues. We determined TGFβs mRNA levels in these tissues and found that only TGFβ2 mRNA was markedly elevated in TNBC than that in luminal BC (Figure 1B and Figure S1A,B). We found that up‐regulation of TGFβ2 mRNA was correlated with down‐regulation of epithelial marker E‐cadherin, and up‐regulation of mesenchymal molecules such as Snail1, N‐cadherin and Vimentin (Figure 1B and Figure S1C) in TNBC. In addition, we measured secreted TGFβs in the culture media (CM) from non‐transformed mammary epithelial MCF10A cells, luminal subtype MCF7, T47D, BT474 and ZR75 cell lines, and TNBC subtype MDA‐MB231, BT549, SUM1315 and Hs578T cell lines. TGFβ2 but not TGFβ1 or TGFβ3 level was much higher in CM of TNBC cell lines than in other cell lines (Figure 1C and Figure S1A,B). Consistently, TNBC cell lines contain high mRNA and protein levels of TGFβ2, gain expression of mesenchymal markers (Snail1 and Vimentin), and lose expression of epithelial markers (ERα and E‐cadherin) (Figure 1D and Figure S1D). To further extend these intriguing observations, we examined TGFβs protein levels in two cohorts of human BC tissues that have a long‐term clinical follow‐up history. In the first cohort, 117 tissues obtained at the Affiliated Cancer Hospital of Guangzhou Medical University contain 68 luminal subtypes and 49 TNBC (Table S2). In the second cohort, 104 tissues obtained at the Sun Yat‐Sen University Cancer Center contain 64 luminal subtypes and 40 TNBC (Table S3). Using immunohistochemistry staining, we found that TGFβ2 and mesenchymal markers were highly expressed in TNBC but not in luminal subtype BC in both cohorts (Figure 1E). Importantly, we found that increased TGFβ2 expression correlated with a worse overall survival in both cohorts (Figure 1F). Together, these data indicate that TGFβ2, but not TGFβ1 or TGFβ3, is overexpressed in TNBC.

### 3.2 TGFβ2 is required for the maintenance of the mesenchymal phenotypes and properties in TNBC

**FIGURE 2:**
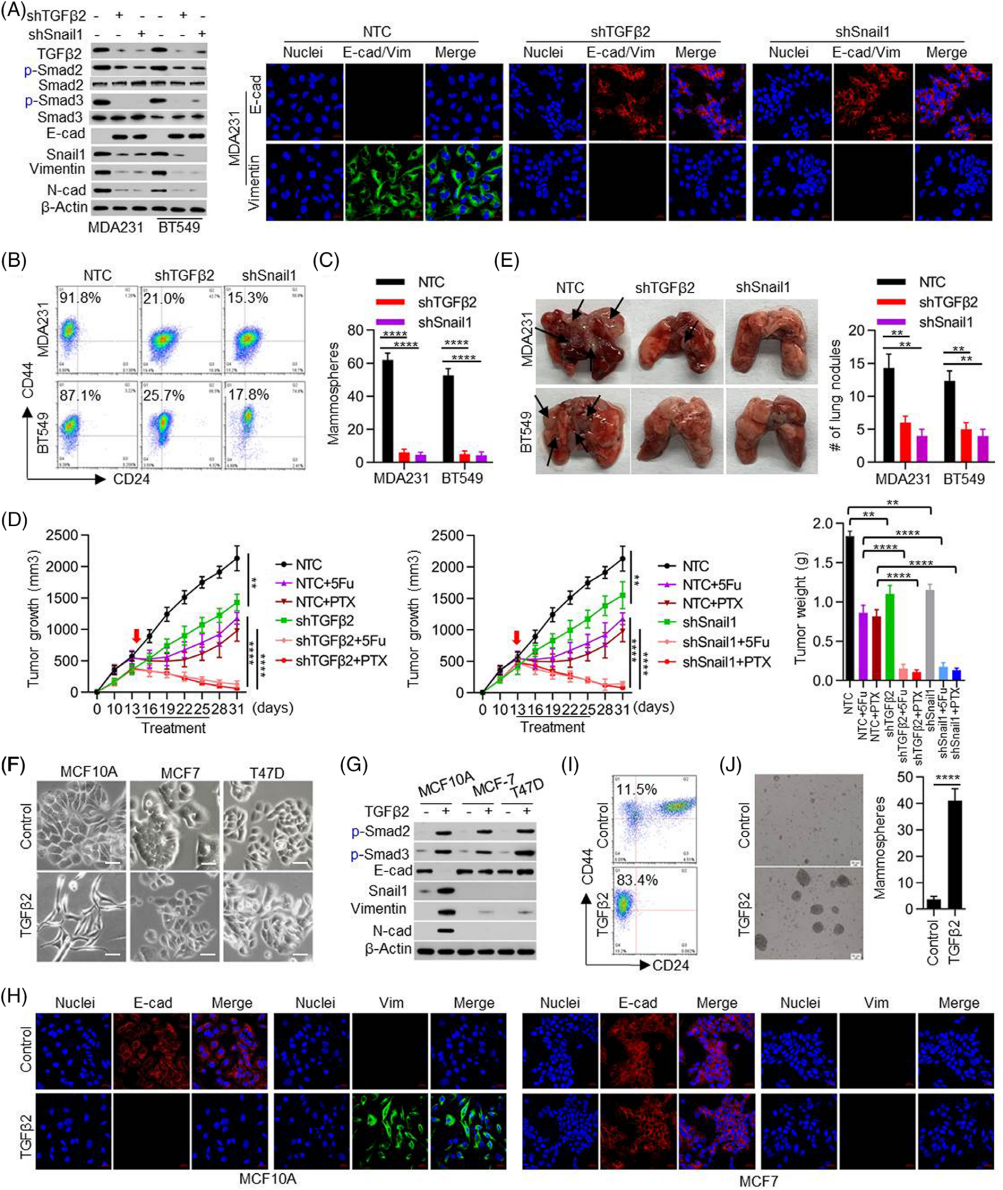
Transforming growth factor‐β2 (TGFβ2)‐Snail1 signalling selectively sustains the mesenchymal‐like traits in triple‐negative BC (TNBC) cells. (A) MDA‐MB231 and BT549 cells with specific knockdown of TGFβ2 or Snail1 by shRNAs. Expression changes of related genes at protein levels were determined by western blot (left) or immunofluorescent staining (right). (B, C) The effects of TGFβ2‐Snail1 on cancer stem cell characteristics were measured with flow cytometry on CD44^high^/CD24^low^ portion and mammosphere forming assay. Two‐sided student's t‐tests were performed. (D) Tumour growth and chemo‐sensitivity in vivo were monitored after TGFβ2 or Snail1 knockdown. Two‐sided student's t‐tests were performed. (E) Metastatic nodules (black arrows) on the lung surface. The number of nodules was quantified on the lungs of nude mice 8 weeks after tail vein injection of related cell lines. Two‐sided student's t‐tests were performed. (F–H) Recombinant human TGFβ2 protein (10 ng/mL, 1 week) induced morphological changes investigated under microscopy and molecule changes as detected by western blot or immunofluorescent staining. (I, J) The effects of recombinant human TGFβ2 protein on cancer stem cell characteristics were measured with flow cytometry on CD44^high^/CD24^low^ portion and mammosphere forming assay. Two‐sided student's t‐tests were performed. The data are presented as the mean ± SEM of at least three independent experiments. **, *p* < .01, ***, *p* < .005, ****, *p* < .0005.

The TGFβ‐Snail1 signalling is critical in mediating EMT and tumour metastasis. To examine whether TGFβ2 is required for the maintenance of EMT phenotype in TNBC, we knocked down TGFβ2 expression in MDA‐MB231 and BT549 cells. As a positive control, we also knocked down Snail1 expression in these cells. Knockdown of TGFβ2 or Snail1 attenuated TGFβ signalling as indicated by the reduction of phosphor‐Smad2 (p‐Smad2) and p‐Smad3 (Figure 2A). TGFβ2‐ or Snail1‐knockdown also induced upregulation of E‐cadherin and downregulation of Snail1, Vimentin and N‐cadherin (Figure 2A and Figure S2A,B). Knockdown of either TGFβ2 or Snail1 reduced the invasive potential (Figure S2C), the population of CD44^high^/CD24^low^ cells (Figure 2B), and the formation of mammosphere (Figure 2C and Figure S2D) of these cells. TGFβ2‐ or Snail1‐knockdown also enhanced the chemo‐sensitivity of these cells to paclitaxel and 5‐Fu in vitro (Figure S2E).

To further evaluate the drug resistance of tumour cells in vivo, MDA‐MB231 cells with either TGFβ2‐ or Snail1‐knockdown were injected into the flank of BALB/c nude mice to form xenograft tumours. After 13 days, mice bearing implanted tumours were treated with phosphate‐buffered saline (PBS), paclitaxel or 5‐Fu every 3 days for six cycles. TGFβ2‐ or Snail1‐knockdown greatly delayed xenograft tumour growth (Figure 2D and Figure S2F). In response to paclitaxel or 5‐Fu treatment, the tumour volume and weight of TGFβ2‐ or Snail1‐knockdown groups decreased significantly more than that of the control group (Figure 2D and Figure S2F). Furthermore, we performed an experimental metastasis analysis by injecting these cells into mice via tail‐vein and found that TGFβ2‐ or Snail1‐knockdown impaired metastatic colonization of MDA‐MB231 and BT549 cell lines (Figure 2E). These data indicate that TGFβ2, similar to Snail1, plays a critical role in maintaining the mesenchymal phenotype, metastatic potential and drug resistance of TNBC cells.

Conversely, we treated non‐transformed mammary epithelial MCF10A cells, luminal BC MCF7 and T47D cells with recombinant human TGFβ2 (Figure 2F,G). Consistent with the previous report, in which TGFβ1‐induced EMT was shown to be a rare event in vitro,^38^ after TGFβ2 treatment over a 20‐day period, only MCF10A but not MCF7 or T47D, cells acquired typical EMT phenotypes with altered expression of EMT markers (Figure 2F‐‐H). Similar observations were found by treating these cells with recombinant human TGFβ1 (Figure S2G). The incapability to induce EMT in MCF7 and T47D cells was not due to the inactivation of the TGFβ signal pathway, because p‐Smad2 and p‐Smad3 were elevated in all these cells after TGFβ2 treatment (Figure 2G). Consistently, TGFβ treatment increased the proportion of CD44^high^/CD24^low^ cell population and the formation of mammosphere in MCF10A cells (Figure 2I,J and Figure S2H), but not in MCF7 or T47D cells (Figure S2I,J). In addition, no significant enhancement of other mesenchymal traits including invasion and anticancer‐drug sensitivity were found in MCF7 or T47D cells after TGFβ treatment (Figure S2K,L).

### 3.3 ERα binds to p‐Smad2/3 to block TGFβ‐Snail1 signalling

**FIGURE 3:**
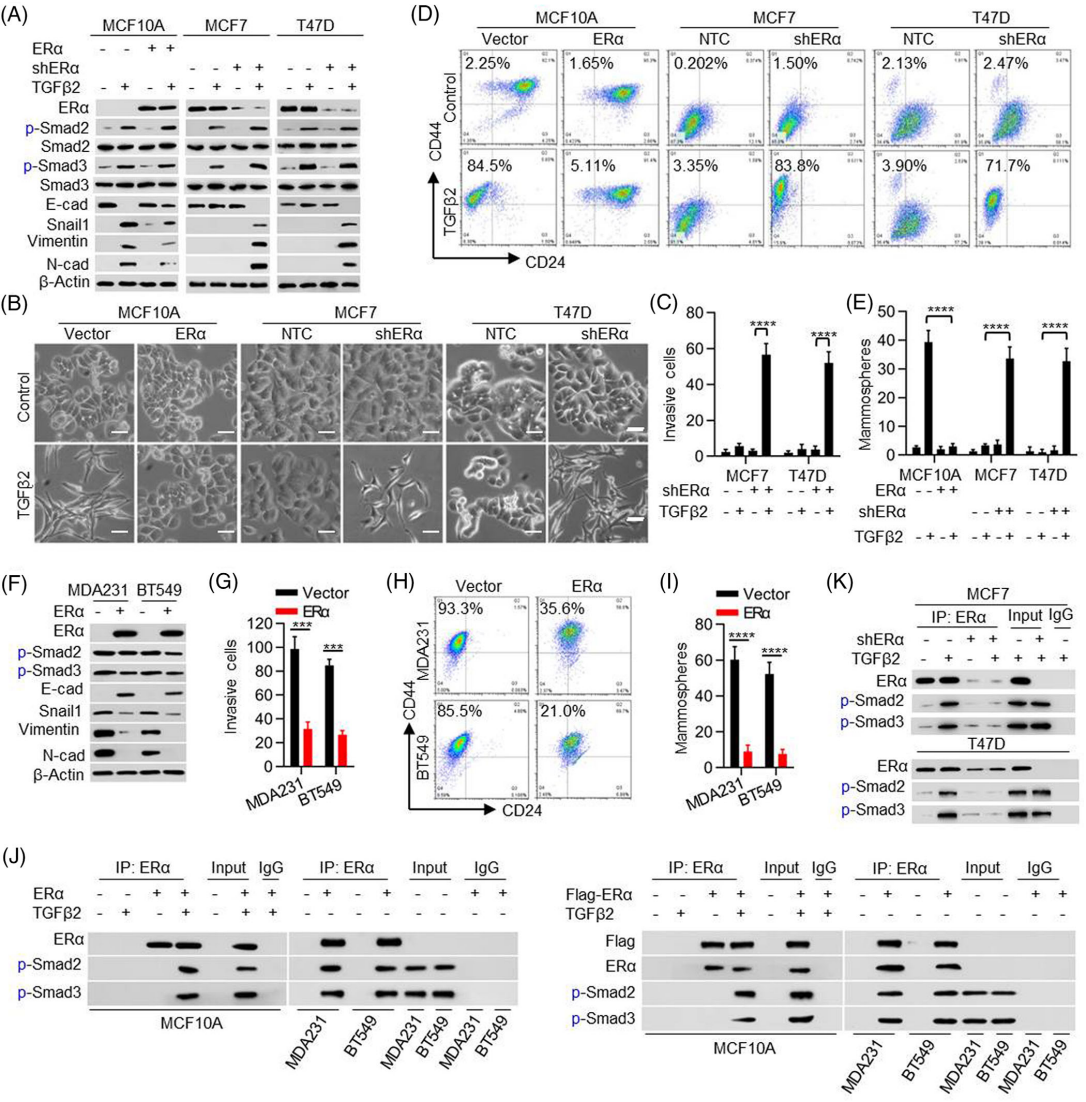
ERα inhibits transforming growth factor‐β2 (TGFβ2)‐Smads‐Snail1 signalling. (A, B) The effects of ERα in recombinant human TGFβ2 protein induced morphological changes were investigated under microscopy and molecule changes as detected by western blot. (C) Cells were transfected with specific ERα shRNA and cultured with or without TGFβ2 (10 ng/mL) treatment. Cellular invasive abilities were detected with transwell assay. Two‐sided student's t‐tests were performed. (D, E) Flow cytometry analysis shows the percentage of CD44^high^/CD24^low^ portion and Mammosphere forming assay used to evaluate the cancer stem cell trait of MCF10A, MCF7 and T47D cells with ERα knockout or overexpression and cultured with or without TGFβ2 (10 ng/mL) treatment. Two‐sided student's t‐tests were performed. (F) Western blot representative images show the expression of ERα, p‐Smad2, p‐Smad3, E‐cadherin, Snail1, Vimentin and N‐cadherin in MDA‐MB231 and BT549 cells with ERα overexpression. (G–I) The effects of ERα overexpression on invasion, CD44^high^/CD24^low^ subpopulation and mammosphere forming were determined in MDA‐MB231 and BT549 cell lines. Two‐sided student's t‐tests were performed. (K) Immunoprecipitation analysis of the interaction between ERα and p‐Smad2/p‐Smad3 in the T47D and MCF7 cells with ERα shRNAs in the presence of TGFβ2 (10 ng/mL) treatment. (J) Immunoprecipitation analysis of the interaction between ERα and p‐Smad2/p‐Smad3 in HMLE, MDA‐MB231 and BT549 cells transfected with ERα or Flag‐ ERα with/without TGFβ2 (10 ng/mL) treatment. The data are presented as the mean ± SEM of at least three independent experiments. ***, *p* < .005, ****, *p* < .0005.

The inability of TGFβ to induce EMT in MCF7 and T47D cells suggests that ERα plays an important role in EMT prevention because MCF7 and T47D cells express ERα whereas MCF10A cells contain no endogenous ERα (Figure 1D). To test this, we knocked down ERα expression in MCF7 and T47D cells (Figure 3A). ERα‐knockdown promoted TGFβ2‐mediated EMT in these cells, accompanied by the downregulation of E‐cadherin, and upregulation of Snail1, Vimentin and N‐cadherin (Figure 3A,B). We performed a co‐immunoprecipitation (CoIP) experiment and found that expressed ERα interacted with Smad2 and Smad3 upon TGFβ2 treatment in MCF7 and T47D cells (Figure S3A). Furthermore, TGFβ2 treatment elevated the invasive potential (Figure 3C and Figure S3B), the population of CD44^high^/CD24^low^ cells (Figure 3D) and the formation of mammosphere (Figure 3E and Figure S3C) in ERα‐knockdown MCF7 and T47D cells.

We also expressed ERα in MCF10A cells and found that ERα significantly blocked TGFβ2‐induced EMT phenotype in those cells (Figure 3A,B). Consistently, ERα expression partially reversed the mesenchymal‐like traits in MDA‐MB231 and BT549 cells, including up‐regulation of E‐cadherin and down‐regulation of Snail1, Vimentin and N‐cadherin (Figure 3F). ERα expression also reduced the invasive potential (Figure 3G and Figure S3D), the population of CD44^high^/CD24^low^ cells (Figure 3H), and the formation of mammosphere in MDA‐MB231 and BT549 cells (Figure 3I and Figure S3E). These findings suggest that ERα expression is able to prevent TGFβs induced EMT in BC cells.

Neither expression nor knockdown of ERα affected p‐Smad2 or p‐Smad3 levels mediated by TGFβ2 but did alter Snail1 expression (Figure 3A,F). We hypothesized that ERα inhibits the TGFβ2‐Smads‐Snail1 signalling at the point of Smad2/3 transactivation. Indeed, ERα expression impaired Smads transactivation in MCF10A cells; and ERα knockdown enhanced Smads transactivation in MCF7 and T47D cells in response to TGFβ2 treatment (Figure 3A and Figure S3F). Furthermore, ERα expression impaired the endogenous Smads transactivation in MDA‐MB231 and BT549 cells (Figure S3G). We also performed a CoIP experiment and found that expressed ERα interacted with p‐Smad2 and p‐Smad3 upon TGFβ2 treatment in MCF10A, MDA‐MB231 and BT549 cells (Figure 3J). Endogenous ERα also interacted with p‐Smad2 and p‐Smad3 in MCF7 and T47D cells in the nucleus (Figure 3K and Figure S3H). However, ERα‐knockdown decreased the interaction between ERα and p‐Smad2/p‐Smad3 in MCF7 and T47D cells in response to TGFβ2 treatment (Figure 3K). These results indicate that ERα inhibits the TGFβ2‐Smads‐Snail1 signalling via interacting with p‐Smad2 and p‐Smad3, and thus suppresses their transcriptional activities, thereby resulting in blocking TGFβ2‐mediated EMT in ERα^+^ luminal cells.

### 3.4 MiR‐141, miR‐145 and miR‐200a target TGFβ2 in BC cells

**FIGURE 4:**
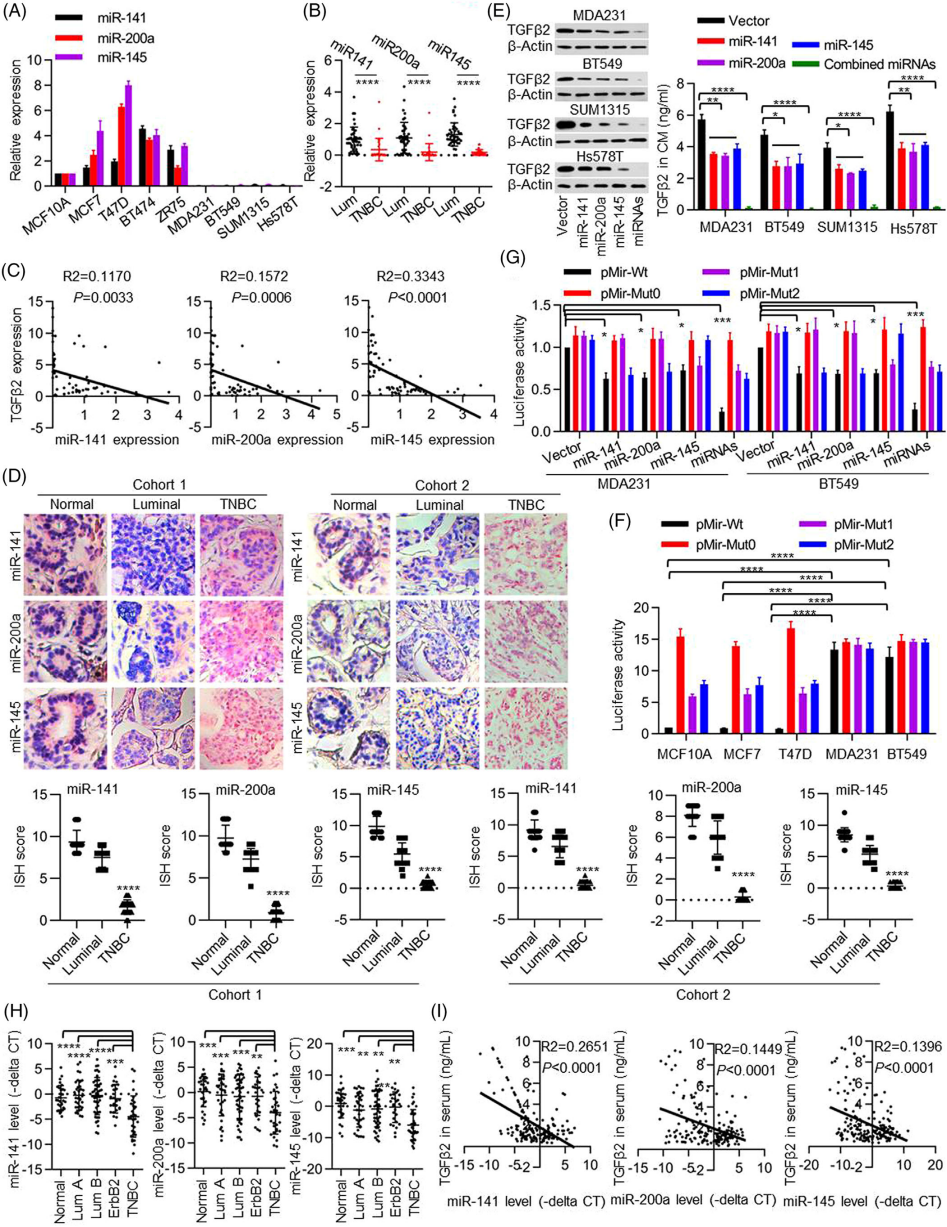
Transforming growth factor‐β2 (TGFβ2) is a direct target of miRNAs_TGFβ2_ in breast cancer cells. (A) miR‐141, miR‐200a and miR‐145 expression in selected cell lines was determined by quantitative reverse‐transcription polymerase chain reaction (qRT‐PCR). (B, C) miRNAs_TGFβ2_ expression in BC tissues was detected by qRT‐PCR and the correlation between TGFβ2 and miRNAs_TGFβ2_ was analyzed. Differences between the two groups were assessed by a two‐way analysis of variance (ANOVA) test. (D) miRNAs_TGFβ2_ expression in two cohorts of BC tissues was examined using ISH assay. The expression of miRNAs is quantitatively and statistically analyzed based on the histologic score. The blue staining represents the miRNA expression level and the red staining represents the nucleus. The histologic score for each section was calculated with the following formula: histologic score  =  proportion score×intensity score. Differences between the two groups were assessed by a two‐way ANOVA test. (E) MDA‐MB231, BT549, SUM1315 and Hs578T cells were transfected with a control vector (Vector) or miRNAs_TGFβ2_ overexpression vector. The expression of TGFβ2 was examined by western blot assays (left). TGFβ2 levels in the CM were detected by enzyme‐linked immunosorbent assay (ELISA) (right). Two‐sided student's t‐tests were performed. (F) The effect of endogenous miRNAs_TGFβ2_ on luciferase activity was observed and luciferase activity driven by pMir‐Wt in MCF10A was set as 1. Two‐sided student's t‐tests were performed. (G) The effect of miRNAs_TGFβ2_ expression interference on luciferase activity was determined and the luciferase activation from cells transfected with pMir‐Wt and interference control were set as 1. Two‐sided student's t‐tests were performed. (H, I) miRNAs_TGFβ2_ levels in serums were determined by qRT‐PCR and the correlation between miRNAs_TGFβ2_ levels and TGFβ2 protein level in serums was analyzed. Differences between the two groups were assessed by a two‐way ANOVA test. The data are presented as the mean ± SEM of at least three independent experiments. *, *p* < .05, **, *p* < .01, ***, *p* < .005, ****, *p* < .0005.

Although the biological roles of TGFβ signalling have been widely studied, the regulation of TGFβ2 expression in cancer remains unclear. We screened for miRNAs that may target TGFβ2 expression via systematic bioinformatics analysis (http://www.targetscan.org) (Table S4). Among the predicted miRNAs with conserved targeting sites against TGFβ2, we found that the expression of miR‐141, miR‐200a and miR‐145 (which we refer to as miRNAs_TGFβ2_) were much lower in MDA‐MB‐231, BT549, SUM1315 and Hs578T cell lines than in MCF10A, MCF7, T47D, BT474 and ZR75 cell lines (Figure 4A). We then determined miR‐141, miR‐200a and miR‐145 levels in a total of 72 fresh‐frozen matched BC and adjacent non‐tumour breast tissues, including 43 luminal type (ERα positive) and 29 TN type BCs (Figure 1B). We found that miR‐141, miR‐200a and miR‐145 (miRNAs_TGFβ2_) levels were significantly lower in TNBC than in ERα^+^ luminal type BC (Figure 4B) and that miRNAs_TGFβ2_ negatively correlated with TGFβ2 level (Figure 4C). We also detected miRNAs_TGFβ2_ in the aforementioned two cohorts of BC tissues via the ISH method and found that miRNAs_TGFβ2_ were lowly expressed in TNBC but highly expressed in normal breast tissue and luminal subtype BC samples (Figure 4D). Most importantly, miR‐141, miR‐200a and miR‐145 negatively correlated with TGFβ2 levels in BC samples (Figure S4A).

To examine whether the miRNAs_TGFβ2_ play key roles in regulating TGFβ2 expression, we constructed recombinant lentiviral vectors expressing miRNAs_TGFβ2_ individually or combinatory (Figure S4B). Transduction of these plasmids significantly up‐regulated miRNAs_TGFβ2_ expression in HEK293T cells (Figure S4C) as well as TNBC cell lines (Figure S4D). Overexpressed miRNAs_TGFβ2_ synergistically down‐regulated intracellular TGFβ2 mRNA expression (Figure S4E) and protein levels as well as in the culture medium (Figure 4E). To further assess whether TGFβ2 is a direct target of miRNAs_TGFβ2_, luciferase reporters linked with TGFβ2‐3′UTR sequences were constructed. These reporters contained both WT sequences with intact miRNAs_TGFβ2_ targeting sequences (pMir‐TGFβ2‐Wt) and those with mutated miRNAs_TGFβ2_ targeting sequences (pMir‐TGFβ2‐Mut). The mutant versions included those with deletion of all miRNAs_TGFβ2_ target regions (pMir‐M0), miR‐141/200a target regions only (pMir‐TGFβ2‐Mut_miR141/200a_ or pMir‐M1) and miR‐145 target region only (pMir‐TGFβ2‐Mut_miR145_ or pMir‐M2). Our results indicate that luciferase reporter activity with WT TGFβ2‐3′UTR was much lower in MCF10A, MCF7 and T47D cell lines, which possess relatively high endogenous levels of miR‐141, miR‐200a and miR‐145 when compared to MDA‐MB231 and BT549 cells (Figure 4F). Expression of miRNAs_TGFβ2_ synergistically inhibited WT luciferase reporter activities in MDA‐MB231 and BT549 cells. In contrast, mutant luciferase reporters lost the inhibitory effect of miRNAs_TGFβ2_ (Figure 4G). In addition, similar synergetic effects of miRNAs_TGFβ2_ on the inhibition of luciferase reporter activities were also found in HEK293T cells (Figure S4F), indicating the importance of the miRNA target regions on TGFβ2‐3′UTR.

To determine whether miRNAs_TGFβ2_ could serve as serum biomarkers, we detected miRNAs_TGFβ2_ in serums from 139 BC patients and 30 healthy controls. After being normalized against spike‐in non‐human miRNA cel‐miR‐67 as a reference,^39^ we found that miRNAs_TGFβ2_ levels were frequently lower in sera from TNBC patients than sera from non‐TNBC patients (Figure 4H) and negatively correlated with TGFβ2 levels in serums (Figure 4I). These results strongly demonstrated that TGFβ2 is a direct target of miRNAs_TGFβ2_.

### 3.5 miRNAs_TGFβ2_ reverse the phenotype of mesenchymal‐like BC cells

**FIGURE 5:**
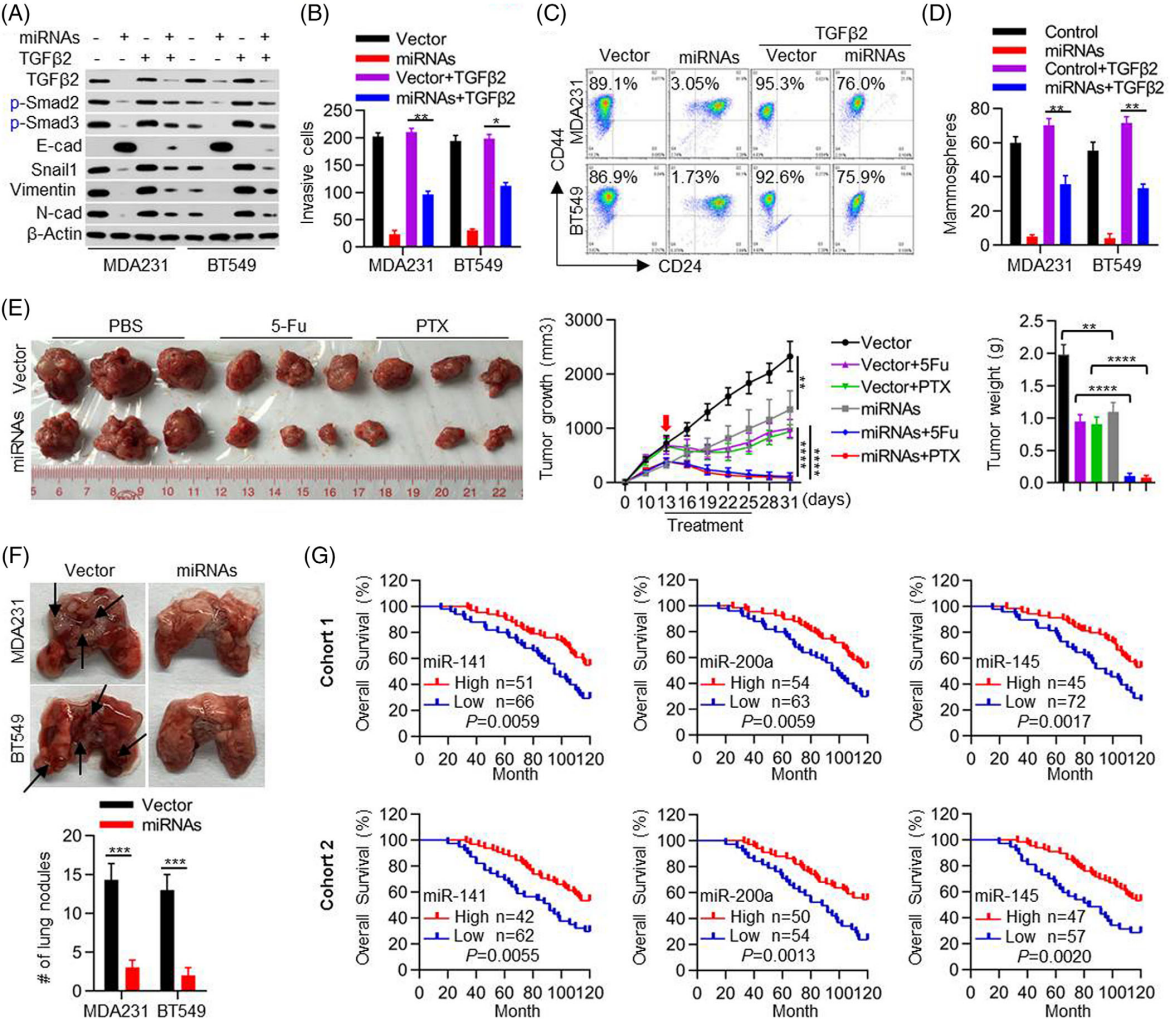
miRNAs_TGFβ2_ reverse the mesenchymal‐like traits in BC cells. (A) The effects of miRNAs_TGFβ2_ in recombinant human transforming growth factor‐β2 (TGFβ2) protein induced the expression of TGFβ2, p‐Smad2, p‐Smad3, E‐cadherin, Snail1, Vimentin and N‐cadherin was detected by western blot. (B) The effects of miRNAs in recombinant human TGFβ2 protein‐induced cell invasion were detected with transwell assay. Two‐sided student's t‐tests were performed. (C) The percentage of CD44^high^/CD24^low^ portion was determined by flow cytometry analysis (D) Mammosphere forming assay was performed to examine the cancer stem cell trait. Two‐sided student's t‐tests were performed. (E) Tumor growth and chemo‐sensitivity in vivo were monitored after miRNAs_TGFβ2_ overexpression. Two‐sided student's t‐tests were performed. (F) Pulmonary metastasis models were constructed with two miRNA overexpression cells or Vector cells (n = 5). Representative photographs of lungs show the metastatic foci (black arrows). Two‐sided student's t‐tests were performed. (G) Kaplan‐Meier analysis estimated overall survival based on miRNAs_TGFβ2_ expression. The histologic score was used to evaluate the expression of miRNAs_TGFβ2_. Survival curves were plotted using the Kaplan‐Meier method and compared using the log‐rank test. *p* < .05 was considered statistically significant. The data are presented as the mean ± SEM of at least three independent experiments. *, *p* < .05, **, *p* < .01, ****, *p* < .0005.

Because miRNAs_TGFβ2_ target TGFβ2 in BC cells, we determined whether miRNAs_TGFβ2_ overexpression has similar effects as TGFβ2 or Snail1 knockdown. By simultaneously overexpressing cell lines with miR‐141, miR‐200a and miR‐145, we found that miRNAs_TGFβ2_ overexpression inhibited TGFβ signalling as exemplified by the down‐regulation of p‐smad2 and p‐smad3, and changed expression levels of EMT markers in MDA‐MB231 and BT549 cells (Figure 5A). Moreover, miRNAs_TGFβ2_ overexpression reduced the invasive potential (Figure 5B and Figure S5A), the percentage of CD44^high^/CD24^low^ population (Figure 5C) and the mammosphere forming ability of TNBC cells (Figure 5D and Figure S5B). Importantly, miRNAs_TGFβ2_ overexpression enhanced the chemo‐sensitivity of MDA‐MB231 and BT549 cells to 5‐Fu and paclitaxel in vitro (Figure S5C). Intriguingly, rescue TGFβ2 treatment reversed the effects of miRNAs_TGFβ2_ on MDA‐MB231 and BT549 cells (Figure 5A‐‐D), indicating that miRNAs_TGFβ2_ exerts their biological roles via repressing TGFβ2 expression.

We then injected miRNAs‐expressing MDA‐MB231 cells into the flank of BALB/c nude mice. After 13 days of implantation, mice were randomly grouped and treated with PBS, 5‐Fu or paclitaxel every 3 days for six cycles. Expression of miRNAs_TGFβ2_ delayed xenograft tumour growth (Figure 5E). In response to 5‐Fu or paclitaxel treatment, xenografts with miRNAs_TGFβ2_ expression decreased in size to a greater extent than that of the control group, indicating a chemo‐sensitizing effect of miRNAs_TGFβ2_ in TNBC cells (Figure 5E). Furthermore, an experimental metastasis assay was performed by injecting tumour cells via the tail‐veil of mice. miRNAs_TGFβ2_ expression inhibited the metastasis of MDA‐MB231 and BT549 cells (Figure 5F). Importantly, we analyzed the correlation of miRNAs_TGFβ2_ expression with patient outcomes in the two BC patient cohorts and found that high miRNAs_TGFβ2_ predicted a better overall survival (Figure 5G). These results clearly indicate that the down‐regulation of miRNAs_TGFβ2_ is mainly responsible for the mesenchymal phenotype and traits observed in TNBC.

### 3.6 Regulation of miRNAs_TGFβ2_ expression by the TGFβ2‐snail1 signalling axis is dependent on EZH2

**FIGURE 6:**
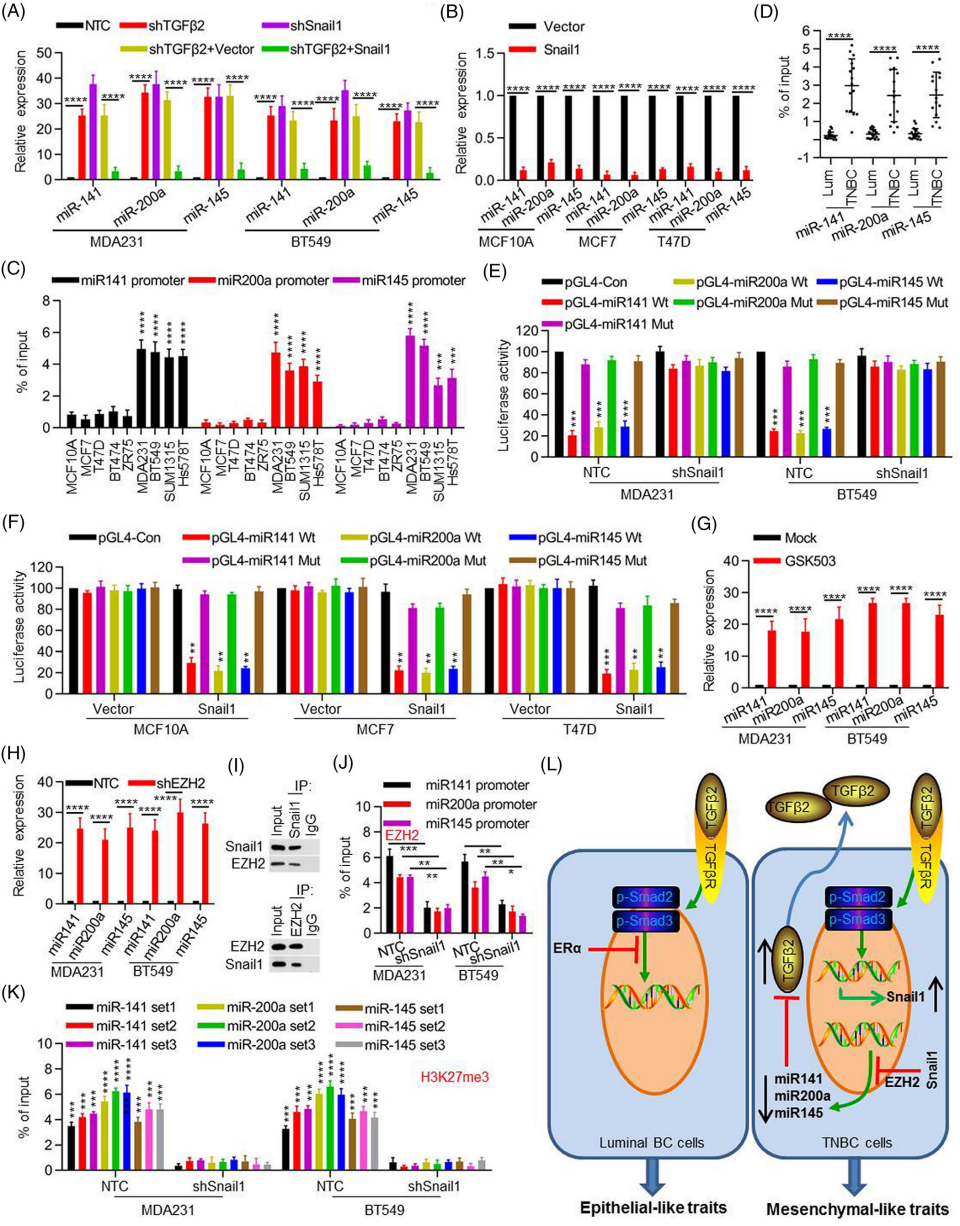
Transforming growth factor‐β2 (TGFβ2)‐Snail1 feedback regulated miRNAs_TGFβ2_ expression. (A) MDA‐MB231 and BT549 cells with specific knockdown of TGFβ2 or/and Snail1 by shRNAs. Then the expression of miR‐141, miR‐200a and miR‐145 was detected using quantitative reverse‐transcription polymerase chain reaction (qRT‐PCR). Two‐sided student's t‐tests were performed. (B) The expression levels of miR‐141, miR‐200a and miR‐145 in Snail1‐overexpressing MCF10A, MCF7 and T47D cells were measured by qRT‐PCR. Two‐sided student's t‐tests were performed. (C, D) The enrichment of Snail1 at the promoters of miR‐141, miR‐200a and miR‐145 in triple‐negative BC (TNBC) cells and tissues was determined by ChIP‐qPCR. Two‐sided student's t‐tests were performed. (E, F) The miR‐141, miR‐200a and miR‐145 promoter‐reporter constructs containing WT or mutant Snail1 binding motif were transfected into Snail1‐knockdown or Snail1‐overexpressing cells. The luciferase activities were measured using a dual luciferase reporter assay kit. Two‐sided student's t‐tests were performed. (G) MDA‐MB231 and BT549 cells were treated with vehicle (Mock) or GSK503. The expression of miR‐141, miR‐200a and miR‐145 were analyzed by qRT‐PCR. Two‐sided student's t‐tests were performed. (H) MDA‐MB231 and BT549 cells were transfected with EZH2 shRNA. The expression of miR‐141, miR‐200a and miR‐145 were detected by qRT‐PCR. Two‐sided student's t‐tests were performed. (I) The interaction between Snail1 and EZH2 in MDA‐MB231 cells was examined by CoIP assay. (J, K) EZH2 binding to miRNAsTGFβ2 promoters and H3K27me3 level at miRNAsTGFβ2 promoters were determined by ChIP‐qPCR. Two‐sided student's t‐tests were performed. (L) A proposed model to illustrate the critical role of TGFβ2‐Snail1‐miRNA_TGFβ2_ Circuitry in BC aggravation. The data are presented as the mean ± SEM of at least three independent experiments. *, *p* < .05, **, *p* < .01, ***, *p* < .005, ****, *p* < .0005.

TGFβ is a cytokine regulating a wide range of physiological and pathophysiological processes via controlling the expression of multiple genes. Given that Snail1‐knockdown repressed TGFβ2 expression (Figure 2A), we explored whether TGFβ2/Snail regulated the expression of miRNAs_TGFβ2_. We found that TGFβ2‐knockdown significantly restored miRNAs_TGFβ2_ expression in TNBC MDA‐MB231 and BT549 cells (Figure 6A). In agreement, Snail1‐knockdown also elevated miRNAs_TGFβ2_ expression in these cells. Consistently, Snail1‐expression abolished the effect of TGFβ2‐knockdown (Figure 6A), suggesting that Snail1 may be responsible for TGFβ2‐mediated miRNAs_TGFβ2_ repression in TNBC cells. Additionally, Snail1‐expression repressed miRNAs_TGFβ2_ expression in MCF10A, MCF7 and T47D cells (Figure 6B). We noticed that there are 4, 4 and 2 consensus Snail1‐binding E‐boxes (CACCTG/CAGGTG) located within the 2 kb region upstream of miR‐141, miR‐200a and miR‐145 precursor transcription start site, respectively (Figure S6A). Chromatin immunoprecipitation‐polymerase chain reaction (ChIP‐PCR) assay showed that endogenous Snail1 was associated at site D for miR‐141, site B for miR‐200a, and sites for miR‐145, respectively in MDA‐MB‐231 and BT549 cells (Figure S6B). ChIP‐qPCR also confirmed that endogenous Snail1 is bound at the promoter of miRNAs_TGFβ2_ in TNBC cells (Figure 6C) and tissues (Figure 6D). To assess whether the 2 kb regions upstream of miRNAs_TGFβ2_ precursor indeed have promoter activity, we cloned the 2 kb DNA into the pGL4 reporter plasmid (as WT) and generated Snail1‐binding site deletion mutants respectively. As shown, luciferase activities driven by miRNAs_TGFβ2_ promoters were much lower in TNBC cells with high endogenous Snail1 expression, compared with those in luminal BC cells. However, these effects were completely abolished on mutant miRNAs_TGFβ2_ promoters (Figure S6C). Additionally, Snail1‐knockdown significantly restored the luciferase activities driven by miRNAs_TGFβ2_ promoters in MDA‐MB231 and BT549 cells (Figure 6E). Consistently, Snail1‐expression decreased miRNAs_TGFβ2_‐promoter luciferase activities in MCF10A, MCF7 and T47D cells (Figure 6F). Interestingly, we noticed that treatment of a selective histone H3K27 methyltransferase EZH2 inhibitor GSK503 enhanced miRNAs_TGFβ2_ expression in MDA‐MB231 and BT549 cells (Figure 6G), and EZH2‐knockdown elevated miRNAs_TGFβ2_ expression (Figure 6H). Although EZH2 was endogenously highly expressed in various BC cell lines (Figure S6D), ChIP‐qPCR assay using the same primers containing Snail1‐binding sites showed the recruitment of EZH2 to the miRNAs_TGFβ2_ promoters in TNBC cells and TNBC tissues (Figure S6E). In line with these observations, increased assembly of Snail1 and EZH2 to miRNAs_TGFβ2_ promoters was accompanied by elevated H3K27me3 in proximity to Snail1‐binding sites at miRNAs_TGFβ2_ promoters (Figure S6F). We hypothesized that Snail1 recruited the EZH2 to miRNAs_TGFβ2_ promoters to repress miRNAs_TGFβ2_ expression. CoIP experiments confirmed the interaction between Snail1 and EZH2 in MDA‐MB231 cells (Figure 6I). Additionally, Snail1‐knockdown abolished the binding levels of EZH2 and the H3K27me3 levels at the miRNAs_TGFβ2_ promoters in MDA‐MB231 and BT549 cells (Figure 6J,K). However, Snail1‐expression enhanced the binding of EZH2 and boosted H3K27me3 levels in MCF10A, MCF7 and T47D cells (Figure S6G). These results indicated that the repression of miRNAs_TGFβ2_ by Snail1 depends on its interaction and recruitment of the EZH2, which results in increased H3K27me3 levels at miRNAs_TGFβ2_ promoters.

## 4 DISCUSSION

In this study, we found that TGFβ2 was the major isoform that was overexpressed in TNBC cell lines, tumour samples and serum. Its expression is required to maintain the mesenchymal traits and aggressive behaviours observed in TNBC. In addition, the TGFβ2/Smad signalling pathway increases the expression of Snail1, which interacts with and recruits EZH2 to silence the expression of miRNAs_TGFβ2_, major endogenous suppressors for TGFβ2 expression. This TGFβ2/Smads‐Snail1/EZH2‐miRNA_TGFβ2_ feed‐forward circuitry is only operated in TNBC, not in luminal subtype BC because ERα interrupts this circuitry by interacting with Smads and blocking Snail1 expression in luminal subtype BC. Our study yields several new insights into the regulation of EMT and provides a plausible cellular differentiation mechanism involved in the development of resistance against endocrine therapy and strategies to prevent BC progression.

First, we found that TGFβ2, but not TGFβ1 or TGFβ3, is preferentially overexpressed in TNBC cell lines, tissues and patient serum. Although TGFβs have three distinct isoforms, they have similar biological activity but have different tissue expression. Mice with TGFβ1‐knockout appeared normal within approximately 3 weeks after birth, but they died soon after due to severe wasting syndrome caused by defects in angiogenesis and hematopoietic function. In addition, TGFβ2‐knockout mice often exhibit developmental defects in the heart, lungs, bones, eyes, and urinary and reproductive systems before birth. TGFβ3‐knockout mice exhibit delayed pulmonary development and palate development defects leading to prenatal death. However, the majority of EMT studies available in the literature are based on TGFβ1, and very little is known about the role of TGFβ2 in EMT. We showed that TGFβ2 expression but not TGFβ1 or TGFβ3 expression is preferentially elevated TNBC cell lines, patient serum and tumour samples. TGFβ2‐knockdown reverses the EMT phenotype, suppresses invasion and CSC properties, and restores chemo‐sensitivity in TNBC, indicating that TGFβ2 is the major isoform of TGFβs involved in maintaining aggressive behaviours in TNBC. Consistent with our findings, a recent study indicated that TGFβ2 is the most potent inducer of EndMT and that TGFβ1‐ and TGFβ3‐induced EndMT indirectly requires TGFβ2 through a paracrine loop.^40^, ^41^ Intriguingly, TGFβ2 can induce the transformation of fibroblasts into myofibroblasts and promote collagen and extracellular matrix (ECM) deposition during wound healing. This phenotype is very similar to a phenotype observed in TNBC cells, which secrete large amounts of ECM during TNBC development and metastasis.^42^, ^43^ Systematic analysis is required to further define the critical role of TGFβ2 in TNBC.

Second, we identified that TGFβ2 expression is tightly regulated by a combination of three miRNAs: miR‐141, miR‐200a and miR‐145 (or miRNAs_TGFβ2_). Although TGFβ can be produced by myeloid cells, mesenchymal cells, and tumour cells via endocrine, paracrine or autocrine manners,^44^ the molecular mechanism that controls TGFβ2 expression remains elusive. miRNAs are small, noncoding RNAs with critical gene‐expression regulatory functions post‐transcriptionally,^45^ functioning as either oncogenes (onco‐miRs) or cancer suppressors.^46^ Additionally, the expression of miRNAs in the blood of cancer patients is different^47^ and serves as tumour biomarkers.^39^, ^48^ Among the predicted miRNAs potentially targeting TGFβ2, we found miRNAs_TGFβ2_ levels were inversely correlated with TGFβ2 levels in TNBC cell lines, patient tumour tissues and serums. Experimental results confirmed that miRNAs_TGFβ2_ synergistically directly repress TGFβ2 expression in TNBC cells. Simultaneous miRNAs_TGFβ2_ overexpression exerts the same biological effects as TGFβ2 knockdown and Snail1 knockdown on changes of CSC proportion, mammosphere formation ability and invasion, and chemotherapy response. The miR‐200 family usually exerts cancer suppressor function by inhibiting the EMT process and CSC phenotype.^49^ miR‐141 down‐regulation closely associated with enhanced cancer growth and metastasis has been reported in several cancer types, such as pancreatic cancer,^50^ renal cell carcinoma^51^ and thyroid cancer.^52^ miR‐200a has been reported to be down‐regulated in cancer and functions as a cancer suppressor via suppressing ZEB1/2 transcription factors to inhibit the EMT.^53^ miR‐200a also can inhibit migration of TNBC cells.^54^ miR‐145 expression is deregulated in several cancer types and its cancer suppressor actions comprise inhibition of cancer growth and metastasis,^55^ induction of apoptosis,^56^ repression of pluripotency^57^ and promotion of differentiation^58^ in stem cells. In consistency with previous reports, our findings supported the cancer suppressor role of miRNAs_TGFβ2_ in BC cells via repressing TGFβ2 expression. However, there are other reports that implied the cancer promoter role of miR‐141 and miR‐200a, comprising enhancement of tumorigenesis^59^ and chemo‐resistance.^60^ These results indicate that the involvement of the miRNAs_TGFβ2_‐TGFβ2 axis in cancer progression is both cancer‐type and cell context‐dependent.

Third, we delineated the detailed molecular mechanism underlying the transcriptional repression of miRNAs_TGFβ2_ by the Snail/EZH2 complex in TNBC. Snail1 usually exerts its transcriptional repressor role via epigenetically modulating chromatin states.^61^, ^62^, ^63^ Here, we further showed that Snail1 interacts with EZH2 and then recruits them to the miRNAs_TGFβ2_ promoter to increase H3K27me3 level. H3K27me3 is a hallmark for gene repression mediated by the Polycomb group of proteins. miRNAs expression mediates a state of equilibrium between gene expression and repression.^64^ Polycomb group proteins play an important role in controlling histone methylation, inducing H3K27.^65^ Overexpression of EZH2 is common in many cancer types because it is involved in cancer initiation, development and progression.^66^ Interestingly, either TGFβ2 or Snail1 knockdown is inversely correlated with H3K27me3 level at miRNAs_TGFβ2_ promoters. Our study revealed that EZH2‐induced H3K27me3 contributes to Snail1‐mediated epigenetic regulation of miRNAs_TGFβ2_ expression to sustain the TGFβ2‐Snail1‐ miRNAs_TGFβ2_ negative regulatory loop in TNBC cells.

Fourth, our study identified the mechanism that explains the activation of EMT in TNBC but not in the luminal BC subtype. TGFβ2 induces EMT in ERα‐ mammary epithelial MCF10A cells but fails to do so in ERα+ MCF7 and T47D cells. In addition, ERα expression blocks TGFβ2‐mediated EMT in MCF10A cells, whereas ERα‐knockdown promotes TGFβ2‐mediated EMT in MCF7 and T47D cells. These findings clearly indicate that ERα is a critical factor in blocking epithelial cells de‐differentiation. ERα reduces Smad protein levels and inhibits TGFβ signalling.^67^ Moreover, ERα interacts with Smad4 to repress estrogen‐induced transcriptional activity.^68^ Our study revealed that ERα interacts with the p‐smad2/3 complex to inhibit the transcription expression of Snail1. ERα is a transcription factor encoded from the *ESR1* gene. Clinically, ERα expression is considered to be a good prognostic marker for BC patients, because ERα is an effective target for anti‐estrogen treatment.^69^ In BC, ERα level generally negatively was correlated with EMT‐TFs such as Twist1, Snail, Slug and ZEB2.^70^ Biologically, ERα signalling promotes mammary epithelia differentiation along a lumina/epithelial lineage by interacting with transcription factor FOXA1.^71^ Functionally in BC, ERα signalling supports growth of the primary lesion, but inhibits metastasis via opposing EMT process. ERα signalling suppresses the expression of EMT‐promoting transcription factors directly or indirectly.^72^ Intriguingly, tamoxifen‐resistant BC cells acquire EMT phenotype and gain CSC properties.^73^ Similarly, long‐term usage of aromatase inhibitors also induces EMT and aggressive phenotype in BC.^74^ These clinical observations are in line with our findings and raise an important issue in endocrine therapy against the luminal subtype of BC. Although ERα is required for the growth and proliferation of luminal subtype of BC, down‐regulation of ERα signalling triggers EMT in tumour cells, leading to drug resistance and increase of metastatic potentials in tumours. EMT is reversible, thus, it will be important to define signalling pathways that contribute to the trigger of EMT and/or maintenance of epithelial state, based on which new therapeutic strategies should be explored. Combinatory targeting of both ERα and EMT signalling (e.g. TGFβ2) may represent an effective approach to enhance the efficiency of BC treatment and prevent invasion and metastasis.

## 5 CONCLUSIONS

In summary, our study identified a new TGFβ2/Smad‐Snail1/EZH2‐miRNATGFβ2 molecular circuit involved in TNBC. The feed‐forward loop is essential for the maintenance of the mesenchymal phenotype and aggressive behaviours of this subtype of BC. Our study also suggested that secreted TGFβ2 could serve as a potential biomarker and druggable target for the detection and treatment of TNBC.

## AUTHOR CONTRIBUTIONS

Liyun Luo, Ying Lin and Guopei Zheng conceived the project. Liyun Luo, Hao Liu and Guopei Zheng designed the study. Liyun Luo, Ning Xu, Weina Fan and Yixuan Wu performed the experiments. Pingping Chen and Ying Lin provided patients’ samples and analyzed the clinical data. Liyun Luo, Ning Xu, Weina Fan, Hao Liu and Guopei Zheng discussed and analyzed the data. Guopei Zheng and Ying Lin wrote the manuscript. All authors reviewed, edited and approved the manuscript.

## CONFLICT OF INTEREST STATEMENT

The authors declare no conflict of interest.

## Supporting information



Supporting Information

Supporting Information
