# Supplementary material for: The TGFβ2‐Snail1‐miRNATGFβ2 Circuitry is Critical for the Development of Aggressive Functions in Breast Cancer
Source: Clin Transl Med. 2024 Feb 1;14(2):e1558. doi: 10.1002/ctm2.1558 (PMC10831563; doi:10.1002/ctm2.1558)
Supplement: Supplementary file 1 — Supporting Information [file CTM2-14-e1558-s002.docx]

**Supplemental Information**

**The TGFβ2-Snail1-miRNA_TGFβ2_ Circuitry is Critical for the Development of Aggressive Functions in Breast Cancer**

Liyun Luo^#^, Ning Xu^#^, Weina Fan^#^, Yixuan Wu, Pingping Chen, Zhihui Li, Zhimin He, Hao Liu^*^, Ying Lin^*^, Guopei Zheng^*^

**Supplementary Data**


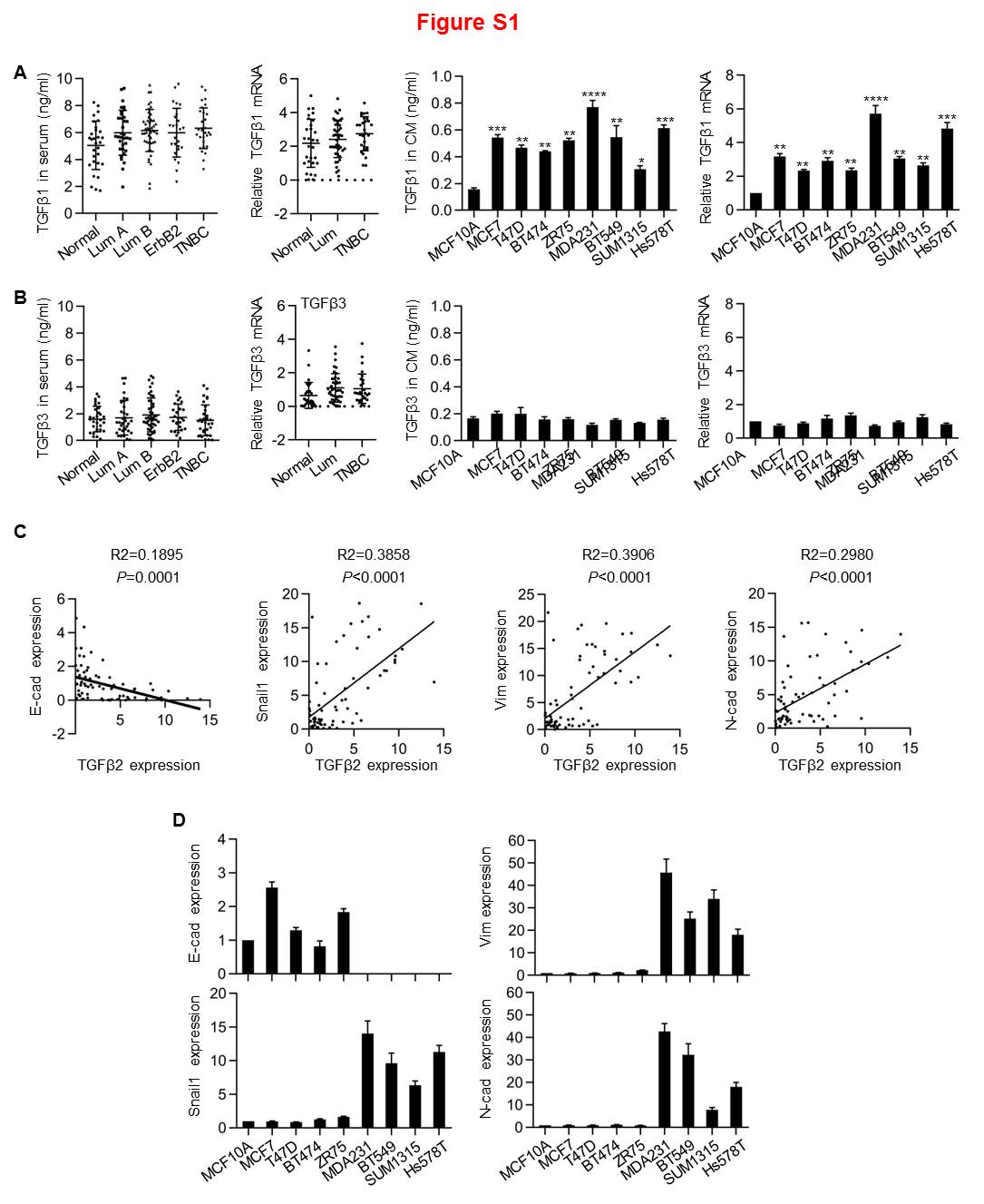


**Figure S1, related to Figure 1.** TGFβ2 expression pattern in BC.

(A and B) TGFβ1 and TGFβ3 protein levels in serum and cell culture medium were determined with ELISA assay. TGFβ1 and TGFβ3 mRNA levels in cell lines and tissues were detected by qRT-PCR.

(C) Correlation analyses between relative TGFβ2 expression and EMT markers expression in the 72 fresh BC tissues.

(D) The expression of E-cadherin, Snail1, Vimentin and N-cadherin in cell lines was examined by qRT-PCR.

The data are presented as the mean ± SEM of at least three independent experiments. *, *P* < 0.05, **, *P* < 0.01, ***, *P* < 0.005, ****, *P* < 0.0005.


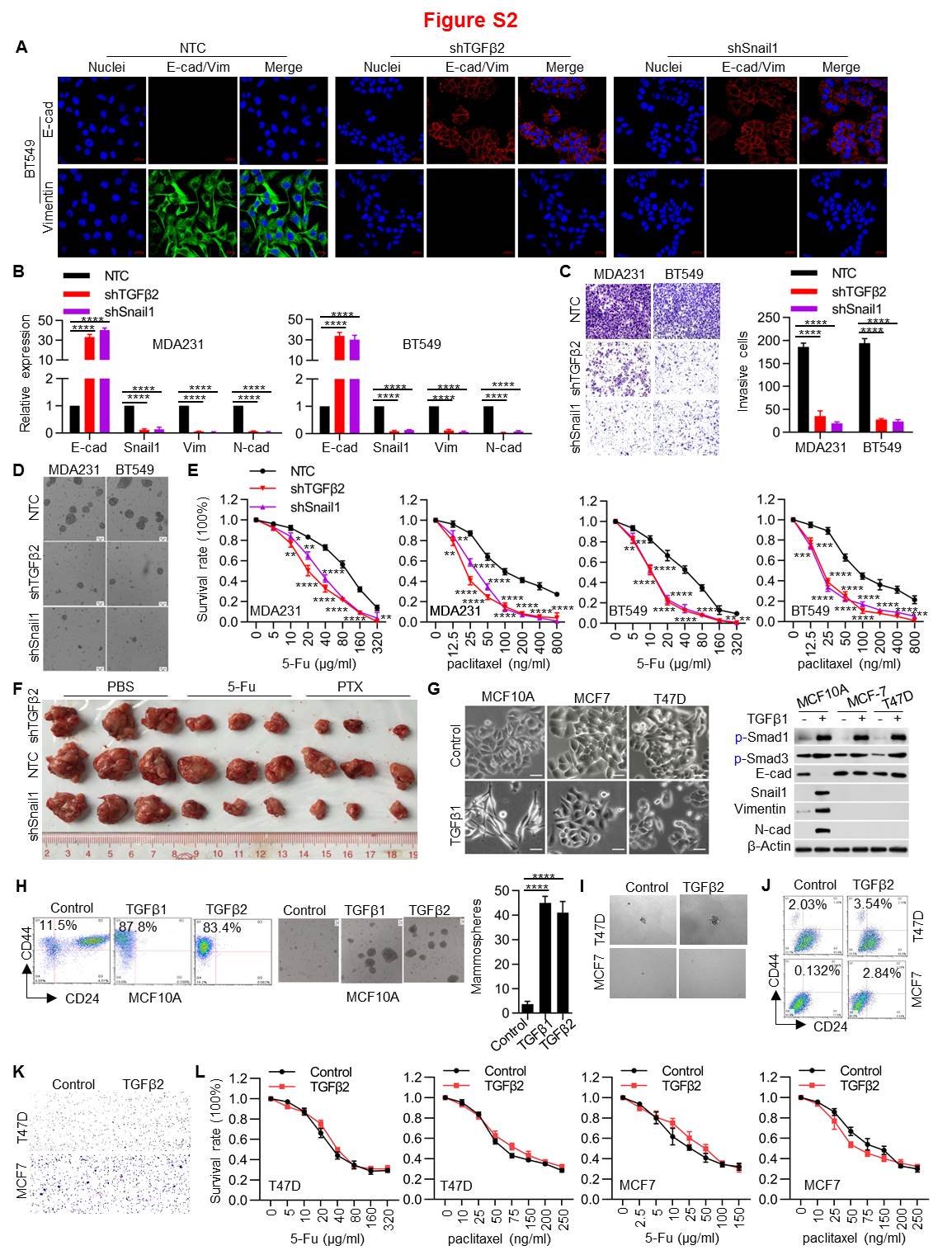


**Figure S2, related to Figure 2.** TGFβ2-Snail1 signaling selectively sustains the mesenchymal-like traits in TNBC cells.

(A) Expression of E-cadherin and Vimentin after TGFβ2 or Snail1 knockdown was analyzed by immunofluorescent staining.

(B) The expression of E-cadherin, Snail1, Vimentin and N-cadherin after TGFβ2 or Snail1 knockdown was detected by qRT-PCR.

(C) Invasion of cells was determined by transwell assay.

(D) The ability of mammosphere forming was examined.

(E) The chemo-sensitivity of BC cells after TGFβ2 or Snail1 knockdown was evaluated using MTS assay.

(F) Tumor growth and chemo-sensitivity in vivo were monitored after TGFβ2 or Snail1 knockdown.

(G) Morphology change and expression change of EMT markers after recombinant human TGFβ1 treatment, examined by western blot.

(H) The effects of TGFβ1 and TGFβ2 on stem cell characteristic in MCF10A cells measured with flow cytometry on CD44^high^/CD24^low^ portion (left) and mammosphere formation assay (right).

(I-J) The effects of TGFβ2 on mammosphere formation assay (left) and stem cell characteristic in MCF7 and T47D cells measured with flow cytometry on CD44high/CD24low portion (right).

(K) Invasion of cells was determined by transwell assay.

(L) The chemo-sensitivity of MCF7 and T47D cells after TGFβ2 treatment was evaluated using MTS assay.

The data are presented as the mean ± SEM of at least three independent experiments. *, *P* < 0.05, **, *P* < 0.01, ***, *P* < 0.005, ****, *P* < 0.0005.


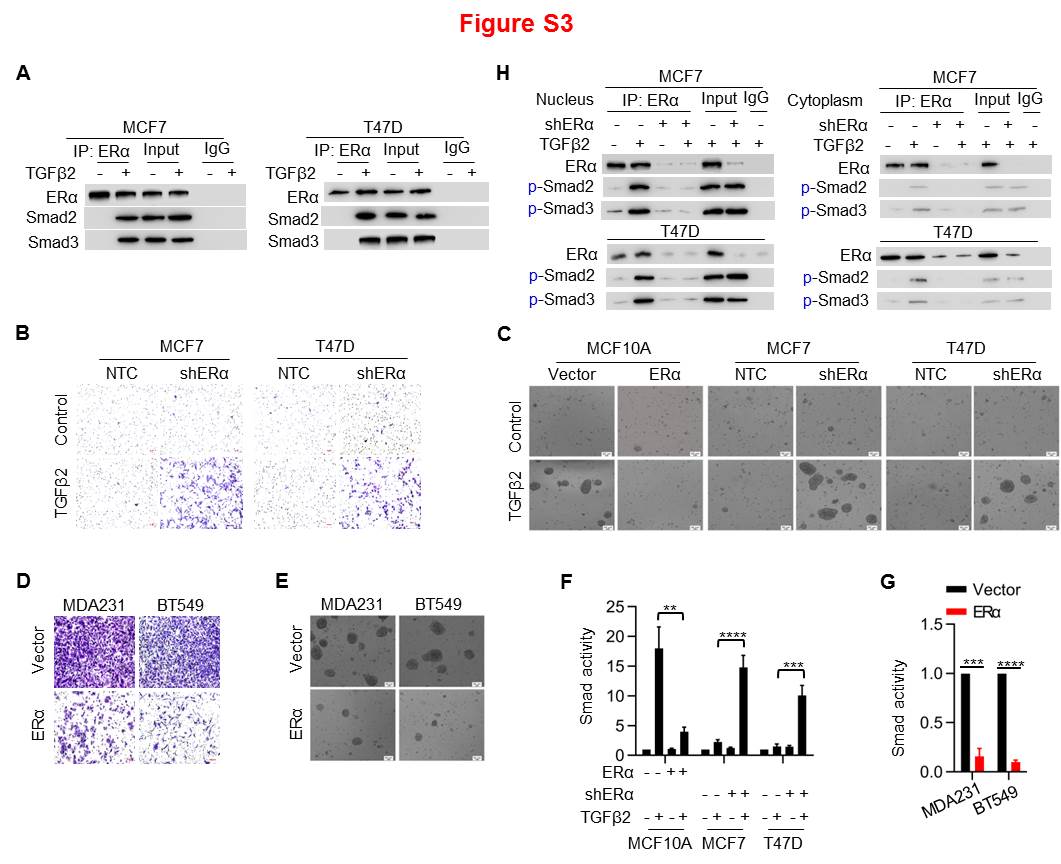


**Figure S3, related to Figure 3.** ERα inhibits TGFβ2-Smads-Snail1 signaling.

(A) Immunoprecipitation analysis of the interaction between ERα and Smad2/Smad3 in the T47D and MCF7 cells with TGFβ2 (10 ng/ml) treatment.

(B) MCF7 and T47D cells with specific knockdown of ERα by shRNAs. Cellullar invasive abilities were detected with transwell assay.

(C) The effect of ERα on recombinant human TGFβ2 protein induced mammosphere forming ability was determined.

(D-E) The effects of ERα on invasion and mammosphere forming were evaluated.

(F-G) Smad2/Smad3 transactivation in related cells was determined with Smads reporter assay.

(H) Immunoprecipitation analysis of the interaction between ERα and p-Smad2/p-Smad3 in the nucleus and cytoplasm of T47D and MCF7 cells with TGFβ2 (10 ng/ml) treatment.

The data are presented as the mean ± SEM of at least three independent experiments. **, *P* < 0.01, ***, *P* < 0.005, ****, *P* < 0.0005.


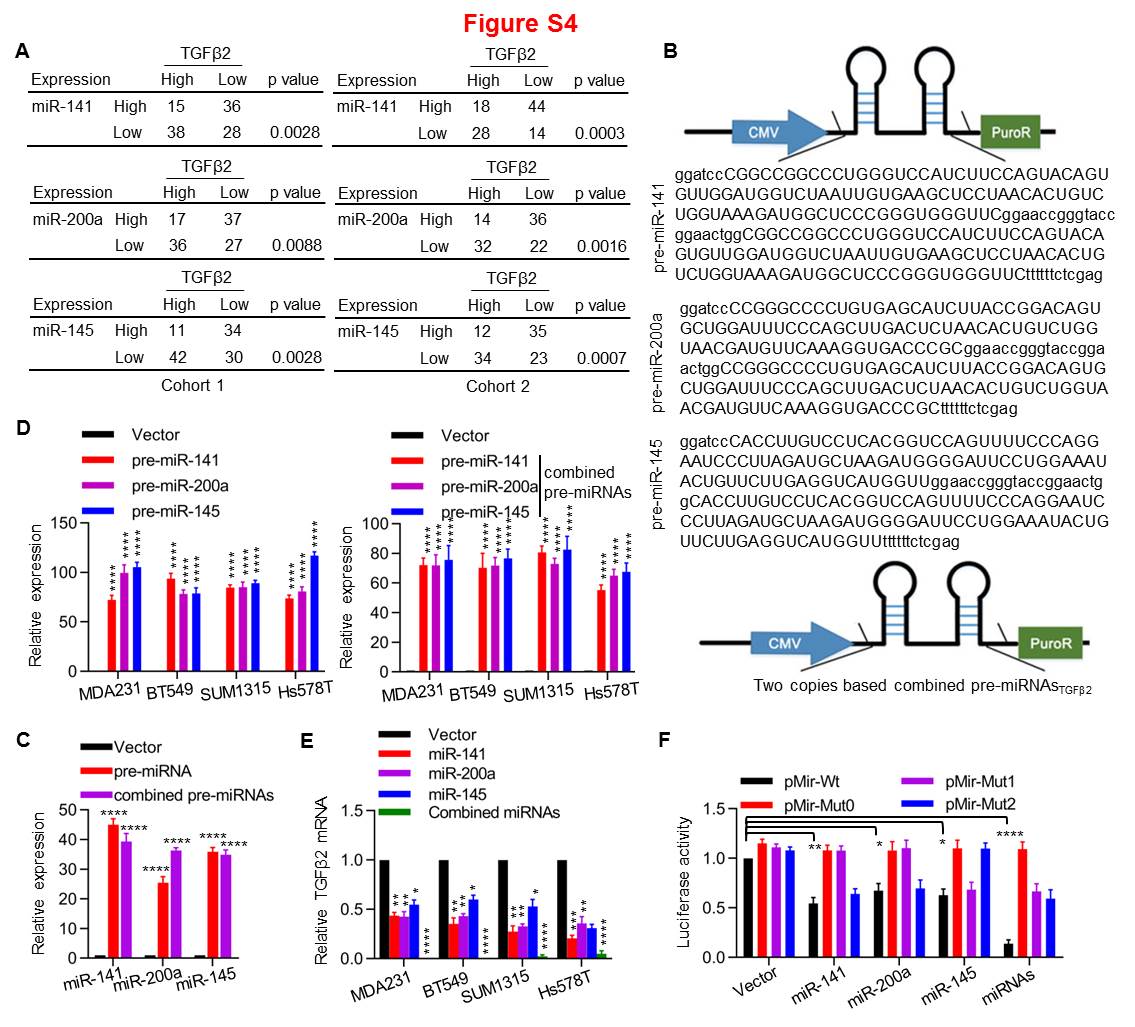


**Figure S4, related to Figure 4.** TGFβ2 is a direct target of miRNAs_TGFβ2_ in BC cells.

(A) Correlation analyses between miRNAs_TGFβ2_ level and TGFβ2 level in the BC tissues, The expression of miRNAs and TGFβ2 is analyzed based on the histologic score. The histologic score for each section was calculated with the following formula: histologic score = proportion score×intensity score.

(B) The structure of miRNAs_TGFβ2_ expression constructs.

(C) Confirmation of miRNAs_TGFβ2_ levels in HEK293T cells after transfection by qRT-PCR.

(D) Confirmation of miRNAs_TGFβ2_ levels in BC cell lines after transfection by qRT-PCR.

(E) qRT-PCR assay was used to confirm miRNAs_TGFβ2_ synergistically down-regulated TGFβ2 mRNA levels in BC cell lines.

(F) miRNAs_TGFβ2_ synergistically inhibited the luciferase activity analyzed in HEK293T cells by luciferase reporter assay.

The data are presented as the mean ± SEM of at least three independent experiments. *, *P* < 0.05, **, *P* < 0.01, ***, *P* < 0.005, ****, *P* < 0.0005.


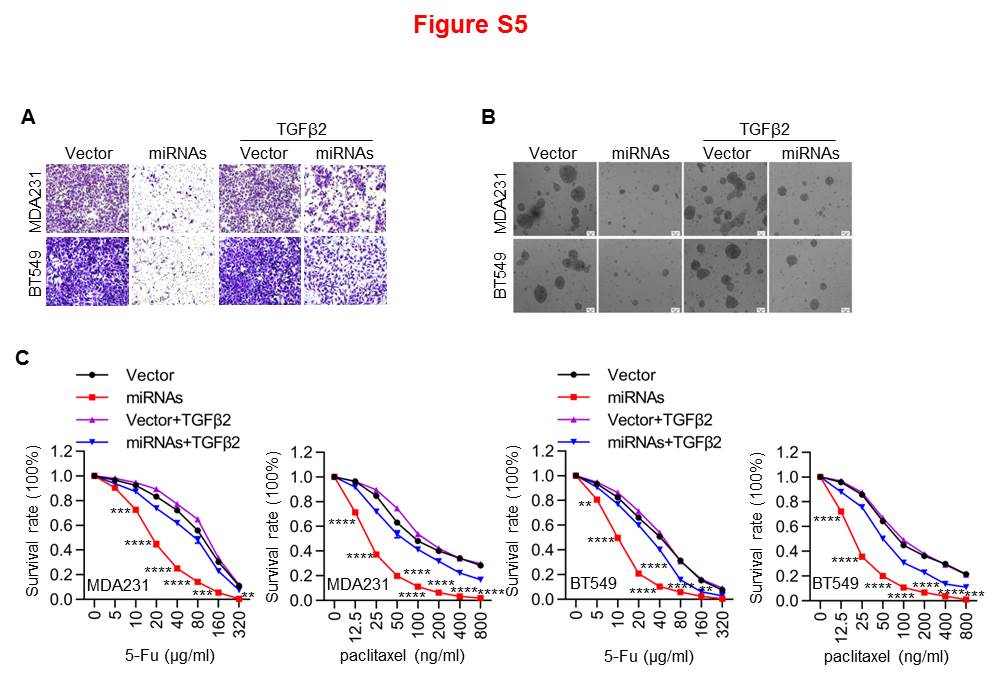


**Figure S5, related to Figure 5.** miRNAs_TGFβ2_ reverse the mesenchymal-like traits in BC cells.

(A) The effects of miRNAs_TGFβ2_ in recombinant human TGFβ2 protein induced cellullar invasive abilities were detected with transwell assay.

(B) The ability of mammosphere forming was examined.

(C) The chemo-sensitivity in vitro was determined by performing MTS assay.

The data are presented as the mean ± SEM of at least three independent experiments. **, *P* < 0.01, ***, *P* < 0.005, ****, *P* < 0.0005.


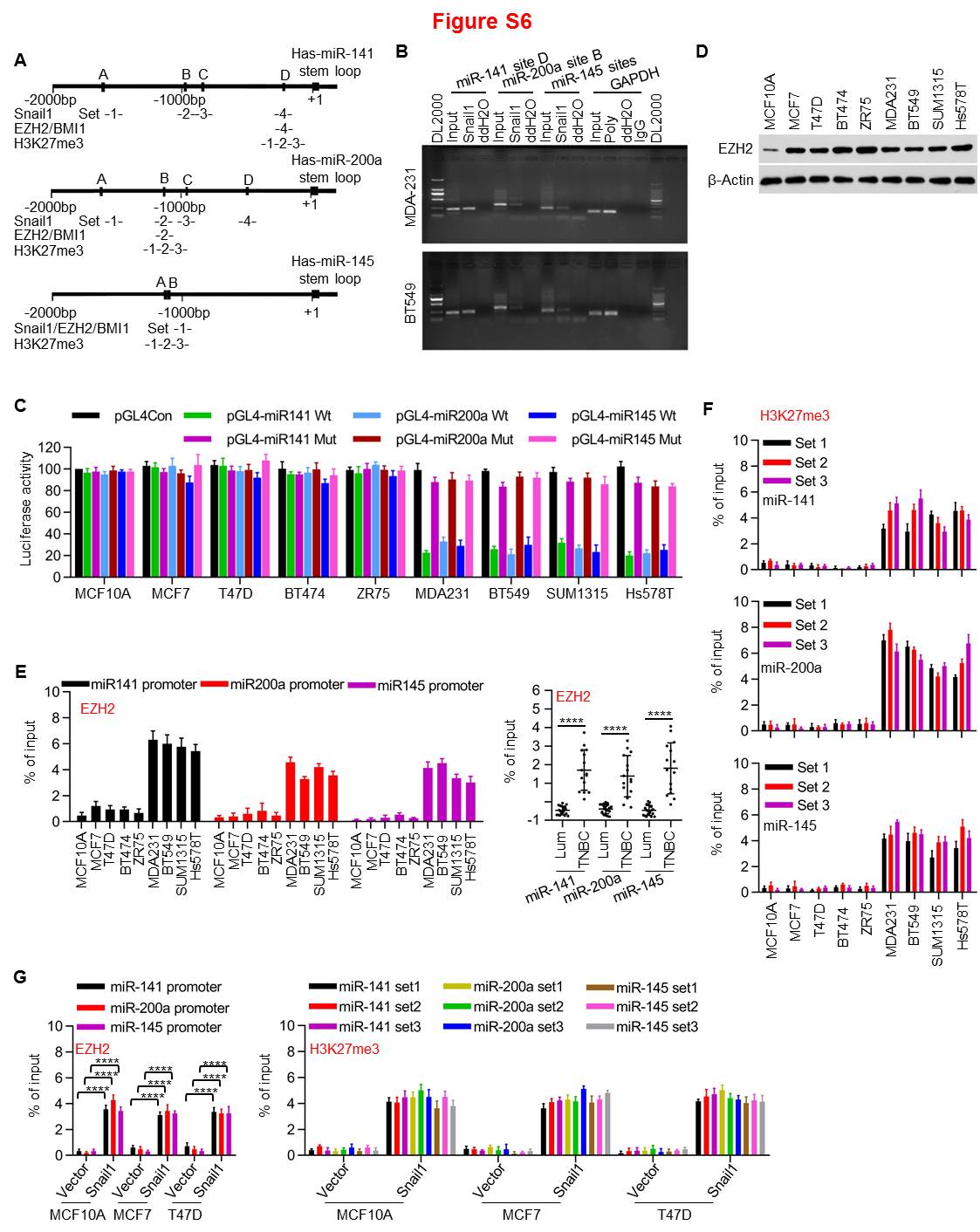


**Figure S6, related to Figure 6.** TGFβ2-Snail1 feedback regulated miRNAs_TGFβ2_ expression.

(A) Schematics representation of Snail1 binding sites in the 2kb putative miRNAs_TGFβ2_ promoters upstream of the base of the miRNAs_TGFβ2_ precursor start site which was set as +1, and the sets of primers used for ChIP are shown.

(B) ChIP-PCR assays identified Snail1 binding sites within the putative miRNAs_TGFβ2_ promoters. The input represents DNA directly after lysis.

(C) Luciferase activity driven by the putative miRNAs_TGFβ2_ promoter in BC cell lines with differential endogenous Snail1 expression was determined.

(D) EZH2 protein levels in selected cell lines examined with western blot.

(E) ChIP-qPCR for EZH2 assembly to miRNAs_TGFβ2_ promoters in BC cell lines and tissues.

(F) ChIP-qPCR for H3K27me3 histone modification at the miRNAs_TGFβ2_ promoters in BC cell lines.

(G) ChIP-qPCR for EZH2 assembly and for H3K27me3 histone modifications at the miRNAs_TGFβ2_ promoters in MCF10A, MCF-7 and T47D cell lines after Snail1 overexpression.

The data are presented as the mean ± SEM of at least three independent experiments. ****, *P* < 0.0005.

**Supplemental Experimental Procedures**

**ELISA assay**

Supernatants of cells grown 48h were collected and centrifuged at 300 g for 5 min. Serum and Cell culture supernatant were assayed for TGFβ1 (SB100C, R&D Systems), TGFβ2 (SB250, R&D Systems) and TGFβ3 (DY243, R&D Systems) levels using commercially available ELISA kits. The experiments for TGFβ1, TGFβ2 and TGFβ3 analysis by ELISA were performed in biological triplicates according to the manufacturer’s instructions.

**Nuclear / Cytoplasmic extraction**

Subcellular fractions (nuclear and cytoplasmic extracts) were performed using the PARIS^TM^ Protein and RNA Isolation System (Invitrogen, AM1921) and according the manufacturer’s recommendations.

**Immunohistochemistry assay**

Formalin-fixed, paraffin-embedded tissue specimens were cut into 4-μm sections. The specimens were deparaffinized in xylene and rehydrated using a series of graded alcohols after being dried at 62°C for 2 hrs. The tissue slides were then treated with 3% hydrogen peroxide in methanol for 15 min. To exhaust endogenous peroxidase activity, and the antigen were retrieved in 0.01 M sodium cirate buffer (pH 6.0) using a microwave oven. After 1 hr of preincubation in 10% goat serum, the specimens were incubated with primary antibody overnight at 4°C with the following primary antibodies: TGFβ2 (MA5-37505, ThermoFisher Scientific), Vimentin (#5741, Cell Signaling Technology), Snail1 (sc-271977, Santa Cruz Biotechnology), N-cadherin (#13116, Cell Signaling Technology), E-cadherin (#3195, Cell Signaling Technology). The tissue slides were treated with a non-biotin horseradish peroxidase detection system according to the manufacturer’s instruction (DAKO, Glostrup, Denmark). The results were scored based on the intensity and the extent of staining. The histologic score for each section was calculated with the following formula: histologic score = proportion score×intensity score. Staining intensity was scored as 0 (negative staining), 1 (weak staining), 2 (moderate staining) and 3 (strong staining). The staining extent was scored based on the percentage of positive cells and was graded as 0 (negative), 1 (0.01–25%), 2 (25.01–50%), 3 (50.01–75%), and 4 (75.01–100%). The total score could be 0, 1, 2, 3, 4, 6, 8, 9, or 12, and the staining could be classified as negative (0, 1, 2, 3, 4) or positive (6, 8, 9, 12). Two different pathologists evaluated the results of IHC.

**Western Blot**

Total protein was extracted from cells using RIPA buffer (Thermo Scientific, Rockford, IL, USA) in the presence of protease inhibitors (Protease Inhibitor Cocktail, Thermo Scientific). The protein concentration of lysates was measured using a BCA Protein Assay Kit (Thermo Scientific). Equivalent amounts of protein were mixed with 5×Lane Marker Reducing Sample Buffer (Thermo Scientific), and resolved by electrophoresis in a 10% SDS–polyacrylamide gel and then transferred onto Immobilon-P Transfer Membrane (Merck Millipore). The membranes were blocked with 5% non-fat milk in Tris-buffered saline and then incubated with the following primary antibodies: TGFβ1 (AF-246-NA), TGFβ2 (MAB612-100) and TGFβ3 (AF-243-NA) antibodies from R&D Systems; E-cadherin (#3195), Snail1 (#3879), Vimentin (#5741), N-cadherin (#13116), EZH2 (#5246), Flag (#14793) and β-Actin (#4967) antibodies from Cell Signaling Technology (Danvers, MA, USA); ERα antibody (sc-8002) from Santa Cruz Biotechnology (Dallas, Texas, USA); phosphor-Smad2 (AB3849-I) and phosphor-Smad3 (07-1389) antibodies from Merck Millipore.

**Immunofluorescence**

Cells were grown on poly-L-lysine-coated glass coverslips (BD Biosciences, San Jose, CA). They were fixed for 15 min with 4% paraformaldehyde in PBS and permeabilized with 0.1% Triton-X100 for 2 min at room temperature, respectively. Coverslips were incubated in blocking solution containing 2% BSA in PBS for 1 h, and incubated at room temperature for 1 h with the appropriate primary antibodies: E-cadherin (#3195, Cell Signaling Technology), Vimentin (#5741, Cell Signaling Technology). After incubation with Alexa Fluor 594-conjugated secondary antibody, cells were stained with DAPI for nuclear staining and then visualized by fluorescence microscopy.

**Transwell assay**

Invasion of HCC cells was assessed using the Cell Invasion Assay Kit (BD Biosciences, Franklin Lakes, NJ, USA) according to the manufacturer’s instructions. Briefly, at 36 h post-transfection, 3×10^4^ cells in 300μl serum-free medium were added to the upper chamber precoated with ECMatrix™ gel. Then, 0.5 ml of 10% FBS-containing medium was added to the lower chamber as a chemoattractant. Cells were incubated for 24h at 37℃, and then non-invading cells were removed with cotton swabs. Cells that migrated to the bottom of the membrane were fixed with pre-cold methanol and stained with 2% Giemsa solution. Stained cells were visualized under a microscope. To minimize the bias, at least three randomly selected fields with 100× magnification were counted, and the average number was taken.

**Mammosphere Assay**

Mammosphere assays were performed as previously described (1). Briefly, single cell suspensions of cell lines were suspended at a density of 20,000 cells/mL in Dulbecco's modified Eagle's medium/F-12 containing 5 mg/mL insulin, 0.5 mg/mL hydrocortisone, 2% B27, and 20 ng/mL epidermal growth factor and seeded into six-well plates with ultra low-attachment surface (2 mL per plate). Mammospheres were counted after 1 to 2 weeks.

**Flow Cytometry Analysis**

The anti-CD44 (clone G44-26) and anti-CD24 (clone ML5) antibodies used for FACS analysis were obtained from BD Bioscience. Briefly, cells were incubated with trypsin–EDTA and dissociated. Cells were pelleted by centrifugation at 500 g for 5 minutes at 4°C, resuspended in 100 μL of monoclonal mouse anti-human CD24-PE antibody and a monoclonal mouse anti-human CD44-APC antibody, and incubated for 20 minutes at 4°C. The sorting was performed following the manufacturer’s instructions. Apoptosis induced by treatment with 100mg/L 5-Fu for 12h was assayed using an AnnexinVFITC/PI Apoptosis Detection Kit (BD Pharmingen, USA) according to standard protocol with FACS analysis.

**Smads Transactivation Reporter Assay**

The Smads transactivation after treatment was determined with the Cignal SMAD Reporter (luc) Kit (SA Biosciences, Frederick, MD, USA). Lipofectamine 2000 (Invitrogen, Carlsbad, CA, USA) was used to transfect cells per well in 96-well plates with 100 ng of the SMAD reporter plasmid. Resulting firefly and Renilla luciferase activities were determined with the DualGlo Luciferase Assay System (Promega). All conditions were measured in triplicates and repeated in an independent experiment. Functionality and transfection efficiency were controlled using the negative and positive control plasmid contained within the Cignal SMAD Reporter Kit.

**Coimmunoprecipitation**

For the co-immunoprecipitation assay, the cells were lysed with modified TNE buffer (50 mM Tris [pH 8.0], 150 mM NaCl, 1% Nonidet P-40 [NP-40], 10 mM sodium fluoride, 10 mM sodium pyrophosphate, 2 mM EDTA) supplemented with 1 mg/L leupeptin, 1 mg/L aprotinin, and 1 mM sodium orthovanadate (Na_3_VO_4_). The immunoprecipitations were performed overnight at 4 °C with antibodies or IgG (as a negative control). The immunoprecipitates were then incubated for 2h with protein G-agarose (Amersham Biosciences, Piscataway, NJ, USA). The reaction products were washed with lysis buffer, and the immune complexes were resolved by SDS-PAGE. Subsequently, western blots were performed.

**miRNA In Site Hybridizations (ISH) assay**

The miR-491-3p expression in tongue cancer samples was detected by In Site Hybridizations (ISH) with kit from Exiqon (Vedbaek Denmark) according to the manufacturer’s instructions as described in our previous study (2). Briefly, the sections were dried at 65°C for 3 h and then deparaffinized in xylene and ethanol at room temperature (RT) followed with a 10 min incubation with proteinase-k at 37°C. After dehydration in ethanol, sections were hybridizated with 40 nM double-DIG LNA™ miR-141, miR-200a or miR-145 probes 55°C for 1 h. After wash in SSC buffer at hybridization temperature and incubation with blocking solution for 15 min, the anti-DIG reagent sheep anti-DIG-AP (Roche, Mannheim, Germany) was applied and incubated for 60 min at RT. After wash in PBST, the sections were incubated with AP substrate NBT-BCIP (Roche) for 2h at 30°C and incubated in KTBT buffer to stop reaction. Then the nuclear counter stain Nuclear Fast Red™ (Vector labs, Burlingame, CA) was applied for 1 min for nuclear counter staining, and slides were rinsed in tap water for 10 min. after dehydrated in ethanol and mounted, the sections were investigated and analyzed under microcopy. The results were scored based on the intensity and the extent of staining. The histologic score for each section was calculated with the following formula: histologic score = proportion score×intensity score. Staining intensity was scored as 0 (negative staining), 1 (weak staining), 2 (moderate staining) and 3 (strong staining). The staining extent was scored based on the percentage of positive cells and was graded as 0 (negative), 1 (0.01–25%), 2 (25.01–50%), 3 (50.01–75%), and 4 (75.01–100%). The total score could be 0, 1, 2, 3, 4, 6, 8, 9, or 12, and the staining could be classified as negative (0, 1, 2, 3, 4) or positive (6, 8, 9, 12). Two different pathologists evaluated the results of IHC.

**Vector construction**

The miR-145-expressing construct, miR-200a-expressing construct, miR-141-expressing construct and control construct were purchased from Genechem (Shanghai, China). Lentiviral shRNA vectors targeting TGFβ2, Snail1, ERα, EZH2 and shRNA scrambled control were ordered from GeneChem (Shanghai, China), and lentivirus particles were generated by cotransfecting the shRNA vectors and packaging plasmids into HEK293T packaging cells. The plasmid for overexpression of ERα (pcDNA3.1) was purchased from GeneChem (Shanghai, China), ShRNA sequences targeting human TGFβ2 were as follows: 5′-CACACTCGATATGGACCAGTT-3′. ShRNA sequences targeting human ERα were as follows: 5′-GCTCATGATCAAACGCTCTAA-3′. ShRNA sequences targeting human Snail1 were as follows: 5′-CCAGGCTCGAAAGGCCTTCAA-3′. ShRNA sequences targeting human EZH2 were as follows: 5′-CCCAACATAGATGGACCAAAT-3′.

**Luciferase reporter assay**

For miRNA luciferase reporter assay: Related DNA sequences from TGFβ2-3’UTR were cloned into pMir-Report plasmid downstream of firefly luciferase reporter gene. Cells were seeded in 96 well-plates and co-transfected with pMir-Report luciferase vector, pRL-TK Renilla luciferase vector and miRNAs_TGFβ2_ expressing vectors using Lipofectamine 2000 (Invitrogen). For promoter activity assay: To determine whether Snail1 regulates the promoter activity of miRNAs_TGFβ2_, potential promoters were cloned into the pGL4-reporter vector upstream of the luciferase gene. Cells were seeded in 96-well plates and co-transfected with the pGL4-reporter vector and the pRL-TK Renilla luciferase vector with or without the Snail1-shRNA vector using Lipofectamine 2000 (Invitrogen). After transfection of 48 h, luciferase activity was determined using a Dual-Luciferase Reporter Assay System (Promega) on the BioTek Synergy 2. The Renilla luciferase activity was used as internal control and the firefly luciferase activity was calculated as the mean ± SD after being normalized by Renilla luciferase activity.

**Total RNA, miRNA and serum miRNA Isolation and qRT-PCR**

Total RNA was isolated from cells and tissues according to the manufacturer’s instructions using RNA isolation Kit (QIAGEN). First-strand cDNA synthesis from total RNA was synthesized with the first-strand synthesis system (Thermo Scientific, Glen Brunie, MA, USA). Real-time PCR was carried out according to standard protocols using an ABI 7500 with SYBR Green detection (Applied Biosystems, Foster City, CA, USA). GAPDH was used as an internal control.

miRNAs from cultured cells were isolated and purified with the miRCURY RNA Isolation Kit (Exiqon, Vedbaek, Denmark). cDNA was generated with the miScript II RT Kit (QIAGEN, Hilden, Germany), and quantitative real-time PCR (qRT-PCR) was performed by using the miScript SYBR Green PCR Kit (QIAGEN) following the manufacturer’s instructions. The miRNA sequence-specific RT-PCR primers and the endogenous control RNU6 were purchased from QIAGEN. The relative quantitative expression was calculated by normalizing the results with RNU6.

Prior to RNA isolation from serum, a *C. elegans* miRNA (cel-miR-67) was spiked into the serum samples with a final concentration of 0.2 nM and used as a reference. Thereafter, 200 μL serum was subjected to miRNA extraction using Biofluids miRCURY RNA Isolation Kit (Exiqon). Then an equal volume of serum miRNA from each participant was reversely transcribed using the miRCURY LNA Universal cDNA synthesis Kit (Exiqon, Vedbaek, Denmark) according to the manufacturer’s instruction. Subsequently, qRT-PCR was performed by using the miScript SYBR Green PCR Kit (QIAGEN) following the manufacturer’s instructions. The levels of miRNA in serum samples were represented as -Δct, where Δct=Ct_target_ – Ct_reference_.

**ChIP-PCR and ChIP-qPCR**

The ChIP assay was performed using the EZ-CHIP^TM^ chromatin immunoprecipitation kit (Merck Millipore). Briefly: Chromatin proteins were cross-linked to DNA by addition of formaldehyde to the culture medium to a final concentration of 1%. After a 10 min incubation at room temperature, the cells were washed and scraped off in ice-cold phosphate-buffered saline (PBS) containing Protease Inhibitor Cocktail II. Cells were pelleted and then resuspended in lysis buffer containing Protease Inhibitor Cocktail II. The resulting lysate was subjected to sonication to reduce the size of DNA to approximately 200–1000 base pairs in length. The sample was centrifuged to remove cell debris and diluted ten-fold in ChIP dilution buffer containing Protease Inhibitor Cocktail II. Samples were kept on ice at all times. A 5 μl sample of the supernatant was retained as “Input” and stored at 4°C. Then 5 µg of **antibodies** were added to the chromatin solution and incubated overnight at 4°C with rotation. After antibody incubation, protein G agarose was added and the sample incubated at 4°C with rotation for an additional 2 h. The protein/DNA complexes were washed with Wash Buffers four times and eluted with ChIP Elution Buffer. Cross-links were then reversed to free DNA by the addition of 5M NaCl and incubation at 65°C for 4 h. The DNA was purified according to the manufacturer’s instructions. 50 μl of DNA was obtained for each treatment. 0.2 μl of DNA from each group was used as a template for PCR. qRT-PCR was carried out according to the standard protocol on ABI 7500 with SYBR Green detection (Applied Biosystems). The results were calculated by normalizing to the positive control, and relative quantization values were calculated using %Input=2% x 2^(Cq 2%Input Sample-Cq IP Sample) method**.** The qPCR primers are listed as follow.

**qRT-PCR primers for Gene Expression**

TGFβ1 (forward: TACAGCACGGTATGCAAGCC; reverse: GCAACCGATCTAGCTCACAGAG), TGFβ2 (forward: CCATCCCGCCCACTTTCTAC; reverse: CCTCGGGCTCAGGATAGTCT), TGFβ3 (forward: ACTTGCACCACCTTGGACTTC; reverse: GGTCATCACCGTTGGCTCA), E-cadherin (forward: GTCAGTTCAGACTCCAGCCC; reverse: AAATTCACTCTGCCCAGGACG), Snail1 (forward: GCTGCAGGACTCTAATCCAGA; reverse: ATCTCCGGAGGTGGGATG), Vimentin (forward: AAAGTGTGGCTGCCAAGAAC; reverse: AGCCTCAGAGAGGTCAGCAA), N-cadherin (forward: GGACAGC CTCTTCTCAATG; reverse: CTGCAGGCTCACTGCTCTC).

**ChIP-qPCR primers**

miR-141 promoter, ChIP-qPCR for Snail1 (set1, forward: GGAGAGAGAACAGCGAGGGATTG, reverse: CAGCCCTCAGTGGAGTCCAAGTC; set2, forward: GGTTGGAGAAAAGAGGCCCCT, reverse: CTCCTTCCTTCTCCGCCTGC; set3, forward: CCTTAAAGCCCCTTCGTCTCCC, reverse: CGCCTCTGAGCCACCTTCCC; set4, forward: CCCCTCGTCTTGAGCTGAGAGC, reverse: GCTCACCAGTTGCTACAGGGGAC); for EZH2 are the same as set4 for Snail1; for H3K27me3 (set1, forward: ACCCAGCAGTGTTTGGGTGCGG, reverse: CTTCCGGGGTGCGTGGGAAGT; set2, forward: CCCCTCGTCTTGAGCTGAGAGC, reverse: CTCAAGGTCGACAGTGGGTTCTGG; set3, forward: ACCCAGTGCGATTTGTCACCTGG, reverse: GGAAGATGGACCCAGGGCCGGCC);

miR-200a promoter, ChIP-qPCR for Snail1 (set1, forward: CCTACAGCCTCTGTGGGTGGAAG, reverse: CTAGGCTTCTACTCCAGGCACACTCAG; set2, forward: CAGACCCCAGACCAGCAGACAC, reverse: GCCCCACGTGCTGCCTTGT; set3, forward: AGAGGGGTGGGGGCAGAGG, reverse: GTGAGCCCCACCCGCCAG; set4, forward: CCCACCAGCATCTCAGAGACCC, reverse: CTCTGGGTTCCACGTGGCTCC); for EZH2 are the same as set2 for Snail1; for H3K27me3 (set1, forward: CAGAGGCGGCTCAGCCCTCGC, reverse: CACTGCACAGCGTCTGAGGGC; set2, forward: GCCCTCAGACGCTGTGCAGTG, reverse: GTTCCAGGAGCTGCTTCCCTG; set3, forward: CAGGGAAGCAGCTCCTGGAAC, reverse: ACCTTCCCTCTGGGTGGTCCC); miR-145 promoter, ChIP-qPCR for Snail1 (forward: CCAGGGTCAAGCAATTCTCGTG, reverse: GCACTTCAAGAATGGATGCCTGG); for EZH2 are the same as set2 for Snail1; for H3K27me3 (set1, forward: GAGACACAGTCTCACTCCATCAC, reverse: TTAGCCAGGTGTGGTGGCGCACAC; set2 is the same as for Snail1; set3, forward: CAGGTGATCTGCCCGCCTCGGCCT, reverse: GATCCACAAATGATGAATGAGTTTC)

**Supplemental reference**

1. Grimshaw MJ, Cooper L, Papazisis K, Coleman JA, Bohnenkamp HR, Chiapero-Stanke L, Taylor-Papadimitriou J, and Burchell JM. Mammosphere culture of metastatic breast cancer cells enriches for tumorigenic breast cancer cells. Breast cancer research : BCR. 2008;10(3):R52.

2. Zheng G, Li N, Jia X, Peng C, Luo L, Deng Y, Yin J, Song Y, Liu H, Lu M, et al. MYCN-mediated miR-21 overexpression enhances chemo-resistance via targeting CADM1 in tongue cancer. *J Mol Med (Berl).* 2016;94(10):1129-41.

**Table S1**

**Clinicopathological variables in 72 breast cancer patients from whom fresh tissues collected**

|  |  | **TGFβ2 expression** | | | |  |
| --- | --- | --- | --- | --- | --- | --- |
| **Factor** | **Cases** | **High** **(≥2 fold)** | | **Low** | | ***P* value** |
| All patients | 72 | 34 | | 38 | |  |
|  |  | No. | % | No. | % |  |
| Age, years |  |  |  |  |  | 0.6287 |
| ≤45 | 27 | 14 | 51.9 | 13 | 48.1 |  |
| ＞45 | 45 | 20 | 44.4 | 25 | 55.6 |  |
| Tumor size (CM) |  |  |  |  |  | 0.0336 |
| ≤2 | 20 | 5 | 25.0 | 15 | 75.0 |  |
| >2 | 52 | 29 | 55.8 | 23 | 44.2 |  |
| Menopause |  |  |  |  |  | 0.8165 |
| Premenopause | 39 | 19 | 48.7 | 20 | 51.3 |  |
| Promenopause | 33 | 15 | 45.5 | 18 | 54.5 |  |
| Node status |  |  |  |  |  | 0.0163 |
| Negative | 18 | 4 | 22.2 | 14 | 77.8 |  |
| Positive | 54 | 30 | 55.6 | 24 | 44.4 |  |
| TNM stage |  |  |  |  |  | 0.0083 |
| I-II | 29 | 8 | 27.6 | 21 | 72.4 |  |
| III- IV | 43 | 26 | 60.5 | 17 | 39.5 |  |
| Distant metastasis |  |  |  |  |  | 0.0019 |
| Negative | 36 | 10 | 27.8 | 26 | 72.2 |  |
| Positive | 36 | 24 | 66.7 | 12 | 33.3 |  |
| ERα status |  |  |  |  |  | ˂0.0001 |
| Negative | 29 | 23 | 79.3 | 6 | 20.7 |  |
| Positive | 43 | 11 | 25.6 | 32 | 74.4 |  |
| E-cadherin |  |  |  |  |  | 0.0086 |
| Low (˂2 fold) | 32 | 21 | 65.6 | 11 | 34.4 |  |
| High | 40 | 13 | 32.5 | 27 | 67.5 |  |
| Snail1 |  |  |  |  |  | ˂0.0001 |
| Low | 34 | 7 | 20.6 | 27 | 79.4 |  |
| High (≥2 fold) | 38 | 27 | 71.1 | 11 | 28.9 |  |
| Vimentin |  |  |  |  |  | ˂0.0001 |
| Low | 35 | 6 | 17.1 | 29 | 82.9 |  |
| High (≥2 fold) | 37 | 28 | 75.7 | 9 | 24.3 |  |
| N-cadherin |  |  |  |  |  | 0.0174 |
| Low | 30 | 9 | 30.0 | 21 | 70.0 |  |
| High (≥2 fold) | 42 | 25 | 59.5 | 17 | 40.5 |  |

**Table S2**

**Clinicopathological variables in 117 breast cancer patients from Cohort1**

|  |  | **TGFβ2 expression** | | | |  |
| --- | --- | --- | --- | --- | --- | --- |
| **Factor** | **Cases** | **High (≥2 fold)** | | **Low** | | ***P* value** |
| All patients | 117 | 53 | | 64 | |  |
|  |  | No. | % | No. | % |  |
| Age, years |  |  |  |  |  | 0.4469 |
| ≤45 | 42 | 17 | 40.5 | 25 | 59.5 |  |
| ＞45 | 75 | 36 | 48.0 | 39 | 52.0 |  |
| Tumor size (CM) |  |  |  |  |  | 0.0154 |
| ≤2 | 36 | 10 | 27.8 | 26 | 72.2 |  |
| >2 | 81 | 43 | 53.1 | 38 | 46.9 |  |
| Menopause |  |  |  |  |  | 0.5806 |
| premenopause | 52 | 22 | 42.3 | 30 | 57.7 |  |
| promenopause | 65 | 31 | 47.7 | 34 | 52.3 |  |
| Node status |  |  |  |  |  | 0.0105 |
| Negative | 24 | 5 | 20.8 | 19 | 79.2 |  |
| positive | 93 | 48 | 51.6 | 45 | 48.4 |  |
| TNM stage |  |  |  |  |  | 0.0046 |
| I-II | 48 | 14 | 29.2 | 34 | 70.8 |  |
| III- IV | 69 | 39 | 56.5 | 30 | 43.5 |  |
| Distant metastasis |  |  |  |  |  | 0.0029 |
| Negative | 58 | 18 | 31.0 | 40 | 69.0 |  |
| Positive | 59 | 35 | 59.3 | 24 | 40.7 |  |
| ERα status |  |  |  |  |  | ˂0.0001 |
| Negative | 49 | 36 | 73.5 | 13 | 26.5 |  |
| positive | 68 | 17 | 25.0 | 51 | 75.0 |  |
| E-cadherin |  |  |  |  |  | 0.0028 |
| Low (˂2 fold) | 66 | 38 | 57.6 | 28 | 42.4 |  |
| High | 51 | 15 | 29.4 | 36 | 70.6 |  |
| Snail1 |  |  |  |  |  | 0.0007 |
| Low | 49 | 13 | 26.5 | 36 | 73.5 |  |
| High (≥2 fold) | 68 | 40 | 58.8 | 28 | 41.2 |  |
| Vimentin |  |  |  |  |  | 0.0078 |
| Low | 27 | 6 | 22.2 | 21 | 77.8 |  |
| High (≥2 fold) | 90 | 47 | 52.2 | 43 | 47.8 |  |
| N-cadherin |  |  |  |  |  | 0.0063 |
| Low | 40 | 11 | 27.5 | 29 | 72.5 |  |
| High (≥2 fold) | 77 | 42 | 54.5 | 35 | 45.5 |  |

**Table S3**

**Clinicopathological variables in 104 breast cancer patients from Cohort2**

|  |  | **TGFβ2 expression** | | | |  |
| --- | --- | --- | --- | --- | --- | --- |
| **Factor** | **Cases** | **High (≥2 fold)** | | **Low** | | ***P* value** |
| All patients | 104 | 46 | | 58 | |  |
|  |  | No. | % | No. | % |  |
| Age, years |  |  |  |  |  | 0.8431 |
| ≤45 | 42 | 18 | 42.9 | 24 | 57.1 |  |
| ＞45 | 62 | 28 | 45.2 | 34 | 54.8 |  |
| Tumor size (CM) |  |  |  |  |  | 0.0206 |
| ≤2 | 33 | 9 | 27.3 | 24 | 72.7 |  |
| >2 | 71 | 37 | 52.1 | 34 | 47.9 |  |
| Menopause |  |  |  |  |  | 0.2274 |
| premenopause | 42 | 22 | 52.4 | 20 | 47.6 |  |
| promenopause | 62 | 24 | 38.7 | 38 | 61.3 |  |
| Node status |  |  |  |  |  | 0.0093 |
| Negative | 19 | 3 | 15.8 | 16 | 84.2 |  |
| positive | 85 | 43 | 50.6 | 42 | 49.4 |  |
| TNM stage |  |  |  |  |  | 0.0019 |
| I-II | 35 | 8 | 22.9 | 27 | 77.1 |  |
| III- IV | 69 | 38 | 55.1 | 31 | 44.9 |  |
| Distant metastasis |  |  |  |  |  | 0.0098 |
| Negative | 47 | 14 | 29.8 | 33 | 70.2 |  |
| Positive | 57 | 32 | 56.1 | 25 | 43.9 |  |
| ER status |  |  |  |  |  | ˂0.0001 |
| Negative | 40 | 35 | 87.5 | 5 | 12.5 |  |
| positive | 64 | 11 | 17.2 | 53 | 82.8 |  |
| E-cadherin |  |  |  |  |  | 0.0030 |
| Low (˂2 fold) | 46 | 28 | 60.9 | 18 | 39.1 |  |
| High | 58 | 18 | 31.0 | 40 | 69.0 |  |
| Snail1 |  |  |  |  |  | 0.0004 |
| Low | 50 | 13 | 26.0 | 37 | 74.0 |  |
| High (≥2 fold) | 54 | 33 | 61.1 | 21 | 38.9 |  |
| Vimentin |  |  |  |  |  | 0.0099 |
| Low | 52 | 16 | 30.8 | 36 | 69.2 |  |
| High (≥2 fold) | 52 | 30 | 57.7 | 22 | 42.3 |  |
| N-cadherin |  |  |  |  |  | 0.0143 |
| Low | 39 | 11 | 28.2 | 28 | 71.8 |  |
| High (≥2 fold) | 65 | 35 | 53.9 | 30 | 46.1 |  |
